# Supplementary material for: A library of avian proteins improves palaeoproteomic taxonomic identification and reveals widespread intraspecies variability
Source: Nat Commun. Author manuscript; Available in PMC 2025 Nov 17. (PMC12494985; doi:10.1038/s41467-025-63886-1)
Supplement: Description of Additional Supplementary Files [file EMS210483-supplement-Description_of_Additional_Supplementary_Files.pdf]

Description of Additional Supplementary Files

Supplementary Data 1. List of genome accessions, source and species of Anatidae included in the study

Supplementary Data 2. Summary of annotated proteins by genome

Supplementary Data 3. Summary of proteins in dataset 1 and dataset 2 by genus. DS1 is Dataset 1 containing sequences 70% complete. DS2 is Dataset 2, containing only unique sequences at the species level

Supplementary Data 4 Summary of inter- and intra-taxon mean distances and intraspecies SAPs

Supplementary Data 5. Genome label index for Figure 1

Supplementary Data 6. Summary of annotation comparison with RefSeq sequences

Supplementary Data 7. Non-Anatidae genomes annotated for the OC116 protein

Supplementary Data 8. Summary of non-Anatidae protein sequences and intraspecies SAPs

Supplementary Data 9. Summary of variant calling for protein-coding regions of the OC116 gene for *Anas platyrhynchos*, source data for figure 5

Supplementary Data 10. PEAKS 11 proteins output for reanalysis of LC-MS/MS data of MC148

Supplementary Data 11. PEAKS 11 peptides output for reanalysis of LC-MS/MS data of MC148

Supplementary Data 12. PEAKS 11 peptide-spectrum match (PSM) output for reanalysis of LC-MS/MS data of MC148

Supplementary Data 13. PEAKS 11 proteins output for reanalysis of LC-MS/MS data of MC123

Supplementary Data 14. PEAKS 11 peptides output for reanalysis of LC-MS/MS data of MC123

Supplementary Data 15. PEAKS 11 peptide-spectrum match (PSM) output for reanalysis of LC-MS/MS data of MC123

Supplementary Data 16. PEAKS 11 proteins output for reanalysis of LC-MS/MS data of MC182

Supplementary Data 17. PEAKS 11 peptides output for reanalysis of LC-MS/MS data of MC182

Supplementary Data 18. PEAKS 11 peptide-spectrum match (PSM) output for reanalysis of LC-MS/MS data of MC182

Supplementary Data 19. PEAKS 11 proteins output for reanalysis of LC-MS/MS data of MC171

Supplementary Data 20. PEAKS 11 peptides output for reanalysis of LC-MS/MS data of MC171

Supplementary Data 21. PEAKS 11 peptide-spectrum match (PSM) output for reanalysis of LC-MS/MS data of MC171

Supplementary Data 22. PEAKS 11 proteins output for reanalysis of LC-MS/MS data of PALTO_114D

Supplementary Data 23. PEAKS 11 peptides output for reanalysis of LC-MS/MS data of PALTO_114D

Supplementary Data 24. PEAKS 11 peptide-spectrum match (PSM) output for reanalysis of LC-MS/MS data of PALTO_114D

Supplementary Data 25. PEAKS 11 proteins output for reanalysis of LC-MS/MS data of PALTO_119D

Supplementary Data 26. PEAKS 11 peptides output for reanalysis of LC-MS/MS data of PALTO_119D

Supplementary Data 27. PEAKS 11 peptide-spectrum match (PSM) output for reanalysis of LC-MS/MS data of PALTO_119D

Supplementary Data 28. PEAKS 11 proteins output for reanalysis of LC-MS/MS data of PALTO_689

Supplementary Data 29. PEAKS 11 peptides output for reanalysis of LC-MS/MS data of PALTO_689

Supplementary Data 30. PEAKS 11 peptide-spectrum match (PSM) output for reanalysis of LC-MS/MS data of PALTO_689

Supplementary Data 31. PEAKS 11 proteins output for reanalysis of LC-MS/MS data of PALTO 693

Supplementary Data 32. PEAKS 11 peptides output for reanalysis of LC-MS/MS data of PALTO_693

Supplementary Data 33. PEAKS 11 peptide-spectrum match (PSM) output for reanalysis of LC-MS/MS data of PALTO_693
