# Supplementary material for: A library of avian proteins improves palaeoproteomic taxonomic identification and reveals widespread intraspecies variability
Source: Nat Commun. Author manuscript; Available in PMC 2025 Nov 17. (PMC12494985; doi:10.1038/s41467-025-63886-1)
Supplement: Reporting Summary [file EMS210483-supplement-Reporting_Summary.pdf]

nature portfolio

Corresponding author(s): Maria C. Codlin
Last updated by author(s): Aug 19, 2025

Reporting Summary

Nature Portfolio wishes to improve the reproducibility of the work that we publish. This form provides structure for consistency and transparency in reporting. For further information on Nature Portfolio policies, see our Editorial Policies and the Editorial Policy Checklist.

Statistics

For all statistical analyses, confirm that the following items are present in the figure legend, table legend, main text, or Methods section.

n/a	Confirmed	
☐	☒	The exact sample size (*n*) for each experimental group/condition, given as a discrete number and unit of measurement
☒	☐	A statement on whether measurements were taken from distinct samples or whether the same sample was measured repeatedly
☒	☐	The statistical test(s) used AND whether they are one- or two-sided<br>*Only common tests should be described solely by name; describe more complex techniques in the Methods section.*
☒	☐	A description of all covariates tested
☒	☐	A description of any assumptions or corrections, such as tests of normality and adjustment for multiple comparisons
☐	☒	A full description of the statistical parameters including central tendency (e.g. means) or other basic estimates (e.g. regression coefficient) AND variation (e.g. standard deviation) or associated estimates of uncertainty (e.g. confidence intervals)
☒	☐	For null hypothesis testing, the test statistic (e.g. *F*, *t*, *r*) with confidence intervals, effect sizes, degrees of freedom and *P* value noted<br>*Give P values as exact values whenever suitable.*
☒	☐	For Bayesian analysis, information on the choice of priors and Markov chain Monte Carlo settings
☒	☐	For hierarchical and complex designs, identification of the appropriate level for tests and full reporting of outcomes
☒	☐	Estimates of effect sizes (e.g. Cohen's *d*, Pearson's *r*), indicating how they were calculated

Our web collection on statistics for biologists contains articles on many of the points above.

Software and code

Policy information about availability of computer code

Data collection	The creation of annotated protein databases employed annotation of a query protein against a target genome and was conducted using NCBI BLAST+ v.2.16.071 and Exonerate v.2.2.072.
Data analysis	MAFFT v.7.45373 was used to align protein segments and the “best-hit” sequence and em_cons from EMBOSS v.6.5.774 was used to create a consensus sequence. Geneious Prime 2024.0 was used to track changes in manual curation of sequences. SRA reads were trimmed with Trimmomatic v.0.39 and alignment to the target gene using the bwa-mem2 v.2.2.1. We performed variant calling using GATK v.4.5.0.0. Analysis of annotated sequences were performed using the `seqinr` package v.4.2-36 and `spider` package v.1.5.0 in Rv.4.4.2, while trees were created using IQTREE v.2.36 and visualized in R using treeio v.1.30.0, tidytree v.0.4.6 and ggtree v.3.14.09 packages. Reanalysis of LC-MS/MS data and identification of peptides was conducted using PEAKS 11 Studio. All methods, programs and packages and code are described in the methods and relevant files and code are uploaded to Zenodo at A description of these files is avaliable at Zenodo 10.5281/zenodo.16932720, as well as in Supplementary Note 5.

For manuscripts utilizing custom algorithms or software that are central to the research but not yet described in published literature, software must be made available to editors and reviewers. We strongly encourage code deposition in a community repository (e.g. GitHub). See the Nature Portfolio guidelines for submitting code & software for further information.

Data

Policy information about availability of data

All manuscripts must include a data availability statement. This statement should provide the following information, where applicable:

- Accession codes, unique identifiers, or web links for publicly available datasets
- A description of any restrictions on data availability
- For clinical datasets or third party data, please ensure that the statement adheres to our policy

All data, curated sequences and related files produced are available in the supplementary files or on Zenodo at 10.5281/zenodo.1693272087. See Supplementary Note 5 for a description of files available on Zenodo. Genomes used in this analysis are publicly available for download from NCBI or the Bird 10,000 Genomes Project website https://b10k.com/index/index/species.html. The complete genome accession list is available in Supplementary Data 1, accessions for Short Read Archives are available in Supplementary Table 1 and for reference proteins accessions are provided in Supplementary Table 5]. LC-MS/MS data used in this article was downloaded from through ProteomeXchange(PXD034547 [[https://proteomecentral.proteomexchange.org/cgi/GetDataset?ID=PXD034547]] and PXD047233 [https://proteomecentral.proteomexchange.org/cgi/GetDataset?ID=PXD047233]). Source Data for Figures 1-5 can be found on Zenodo at 10.5281/zenodo.1693272087, as well as in the Supplementary Data 4 for Figure 3 and Supplementary Data 9 for Figure 5.

Research involving human participants, their data, or biological material

Policy information about studies with human participants or human data. See also policy information about sex, gender (identity/presentation), and sexual orientation and race, ethnicity and racism.

Reporting on sex and gender	*Use the terms sex (biological attribute) and gender (shaped by social and cultural circumstances) carefully in order to avoid confusing both terms. Indicate if findings apply to only one sex or gender; describe whether sex and gender were considered in study design; whether sex and/or gender was determined based on self-reporting or assigned and methods used. Provide in the source data disaggregated sex and gender data, where this information has been collected, and if consent has been obtained for sharing of individual-level data; provide overall numbers in this Reporting Summary. Please state if this information has not been collected. Report sex- and gender-based analyses where performed, justify reasons for lack of sex- and gender-based analysis.*
Reporting on race, ethnicity, or other socially relevant groupings	*Please specify the socially constructed or socially relevant categorization variable(s) used in your manuscript and explain why they were used. Please note that such variables should not be used as proxies for other socially constructed/relevant variables (for example, race or ethnicity should not be used as a proxy for socioeconomic status). Provide clear definitions of the relevant terms used, how they were provided (by the participants/respondents, the researchers, or third parties), and the method(s) used to classify people into the different categories (e.g. self-report, census or administrative data, social media data, etc.) Please provide details about how you controlled for confounding variables in your analyses.*
Population characteristics	*Describe the covariate-relevant population characteristics of the human research participants (e.g. age, genotypic information, past and current diagnosis and treatment categories). If you filled out the behavioural & social sciences study design questions and have nothing to add here, write "See above."*
Recruitment	*Describe how participants were recruited. Outline any potential self-selection bias or other biases that may be present and how these are likely to impact results.*
Ethics oversight	*Identify the organization(s) that approved the study protocol.*

Note that full information on the approval of the study protocol must also be provided in the manuscript.

Field-specific reporting

Please select the one below that is the best fit for your research. If you are not sure, read the appropriate sections before making your selection.

☒ Life sciences ☐ Behavioural & social sciences ☐ Ecological, evolutionary & environmental sciences

For a reference copy of the document with all sections, see nature.com/documents/nr-reporting-summary-flat.pdf

Life sciences study design

All studies must disclose on these points even when the disclosure is negative.

Sample size	All genomes available on as of February 2024 on NCBI and through the Bird 10,000 genome project for Anatidae taxa were considered for the study. 173 genomes were downloaded and 13 of these were excluded.
Data exclusions	9 Genomes were excluded as no high quality protein sequences were recovered from them, all belonged to Cygnus atratus. Four genomes were also excluded from the analysis as their protein alignments indicated potential misidentifications of the sample species: D2006016059 ("Anas acuta"), DT2207277613-1 ("Mareca penelope"), D2104051175 ("Mareca sibilatrix"), and D2104051303 ("Merganetta armata"). All excluded samples are listed in supplementary data 1.

Replication	The code avaliable on Zenodo will reproduce the same uncurated annotations, assuming the same version of the genome is employed. Multiple versions of the database were created, and when the inputs remains (i.e. the code, target and query) the same, the annotated protein sequences remained the same. All manual changes to the uncurated sequences were tracked using Geneious Prime 2024.0 and are avaliable in the "Complete dataset.zip" on Zenodo and can be used to replicate curation steps. Figures and analysis from this paper can be reproduced using the curated dataset provided in Dataset1.zip, and the code made avaliable on Zenodo. The phylogenetic analysis employed 1000 bootstrap replicates and is reflected in the bootstrap score.
Randomization	Randomisation was employed for bootstrap replication in phylogenetic analysis. For the remaining analysis, randomization was not relevant to this project as we are examining a single variable and not testing statistical differences among groups.
Blinding	Blinding was not relevant to this project as we were not testing statistical differences among groups.

Reporting for specific materials, systems and methods

We require information from authors about some types of materials, experimental systems and methods used in many studies. Here, indicate whether each material, system or method listed is relevant to your study. If you are not sure if a list item applies to your research, read the appropriate section before selecting a response.

Materials & experimental systems

n/a	Involved in the study
☒	☐ Antibodies
☒	☐ Eukaryotic cell lines
☒	☐ Palaeontology and archaeology
☒	☐ Animals and other organisms
☒	☐ Clinical data
☒	☐ Dual use research of concern
☒	☐ Plants

Methods

n/a	Involved in the study
☒	☐ ChIP-seq
☒	☐ Flow cytometry
☒	☐ MRI-based neuroimaging

Plants

Seed stocks	*Report on the source of all seed stocks or other plant material used. If applicable, state the seed stock centre and catalogue number. If plant specimens were collected from the field, describe the collection location, date and sampling procedures.*
Novel plant genotypes	*Describe the methods by which all novel plant genotypes were produced. This includes those generated by transgenic approaches, gene editing, chemical/radiation-based mutagenesis and hybridization. For transgenic lines, describe the transformation method, the number of independent lines analyzed and the generation upon which experiments were performed. For gene-edited lines, describe the editor used, the endogenous sequence targeted for editing, the targeting guide RNA sequence (if applicable) and how the editor was applied.*
Authentication	*Describe any authentication procedures for each seed stock used or novel genotype generated. Describe any experiments used to assess the effect of a mutation and, where applicable, how potential secondary effects (e.g. second site T-DNA insertions, mosiacism, off-target gene editing) were examined.*
