# Supplementary material for: A library of avian proteins improves palaeoproteomic taxonomic identification and reveals widespread intraspecies variability
Source: Nat Commun. Author manuscript; Available in PMC 2025 Nov 17. (PMC12494985; doi:10.1038/s41467-025-63886-1)
Supplement: Supplementary Information [file EMS210483-supplement-Supplementary_Information.pdf]

Supplementary Information

for

A library of avian proteins improves palaeoproteomic taxonomic identification and reveals widespread intraspecies variability

Maria C. Codlin[1*], Lisa Yeomans[2,3], Josefin Stiller[4], Beatrice Demarchi[1]

[1] Department of Life Sciences and Systems Biology, University of Turin, Turin, Italy
[2] Globe Institute, Section for GeoBiology, University of Copenhagen, Copenhagen, Denmark
[3] Institute of Archaeology, University College London, London, United Kingdom
[4] Department of Biology, University of Copenhagen, Denmark

*Corresponding author: Maria Codlin (maria.codlin@unito.it)

Supplementary Figures

Supplementary Figure 1. Phylogenetic tree derived from 13 concatenated protein sequences for 160 Anatidae genomes. Samples are coloured by genus. The tree was rooted with the outgroup *Grus americana* (whooping crane), which is not shown in the plot. Bootstrap resampling support shown for each node opaque grey circle = >90% and semi-transparent grey circle =80-90%, transparent circle <80%. The tree was rooted with the outgroup *Grus americana* (whooping crane), which is not shown in the plots. Source data available on Zenodo[1].

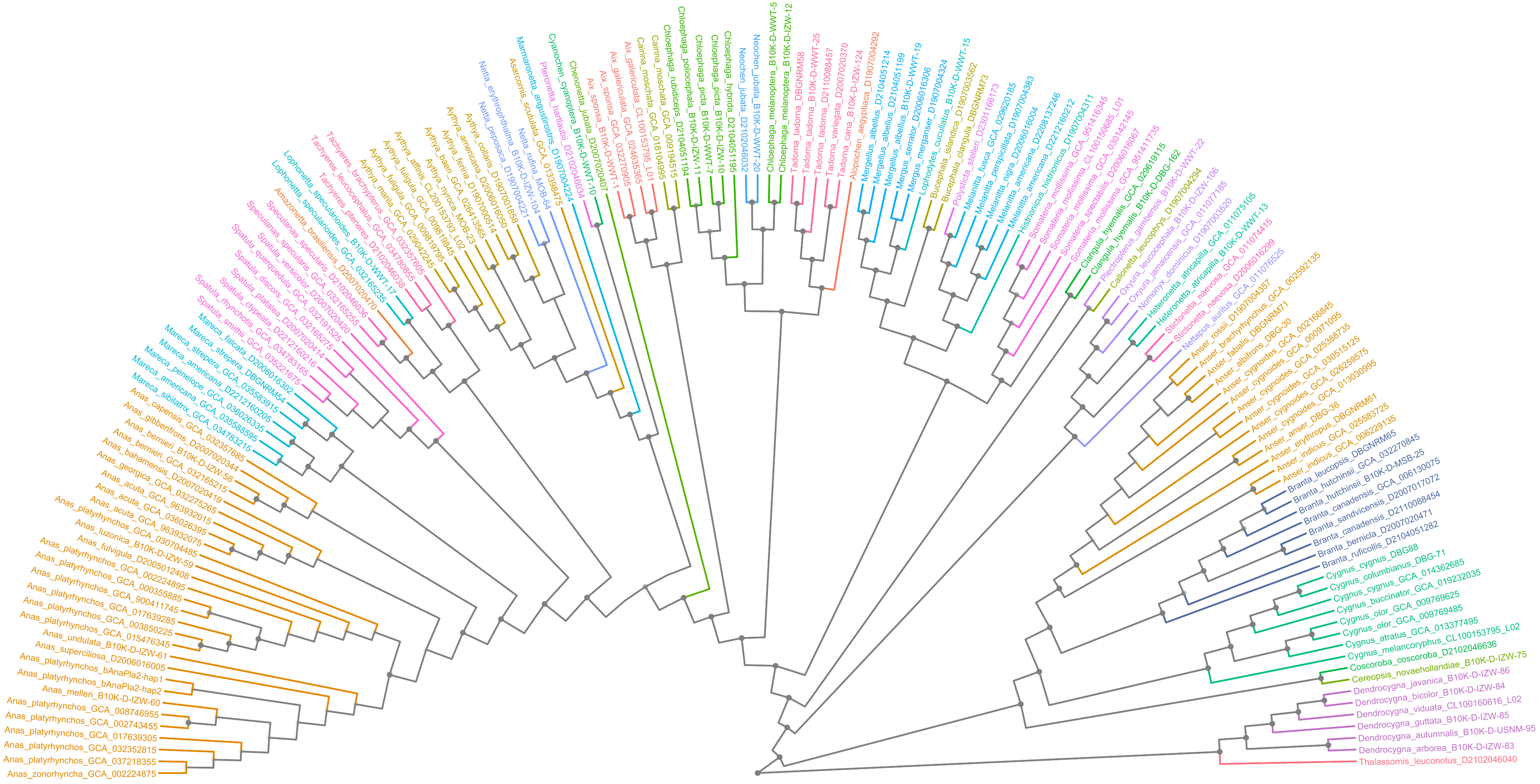

Supplementary Figure 2. Phylogenetic tree derived from 160 Anatidae Albumin sequences. The tree was rooted with the outgroup *Grus americana* (whooping crane), not shown in the plot. Bootstrap resampling support shown for all nodes. Source data: Zenodo[1].

Albumin

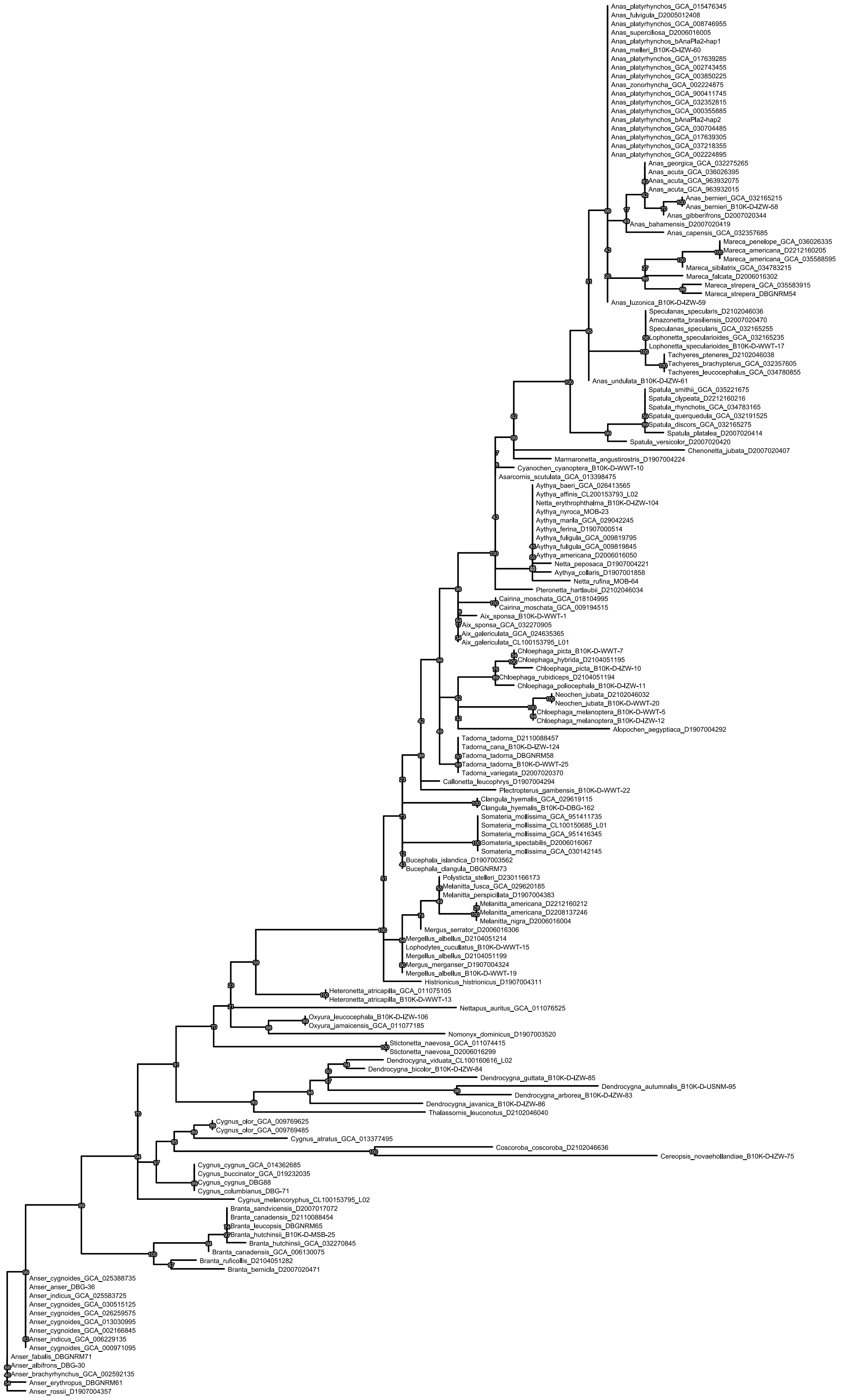

Supplementary Figure 3. Phylogenetic tree derived from 157 BPIfoldB4 sequences. The tree was rooted with the outgroup *Grus americana* (whooping crane), not shown in the plot. Bootstrap resampling support shown for all nodes. Source data: Zenodo[1].

BPIfoldB4

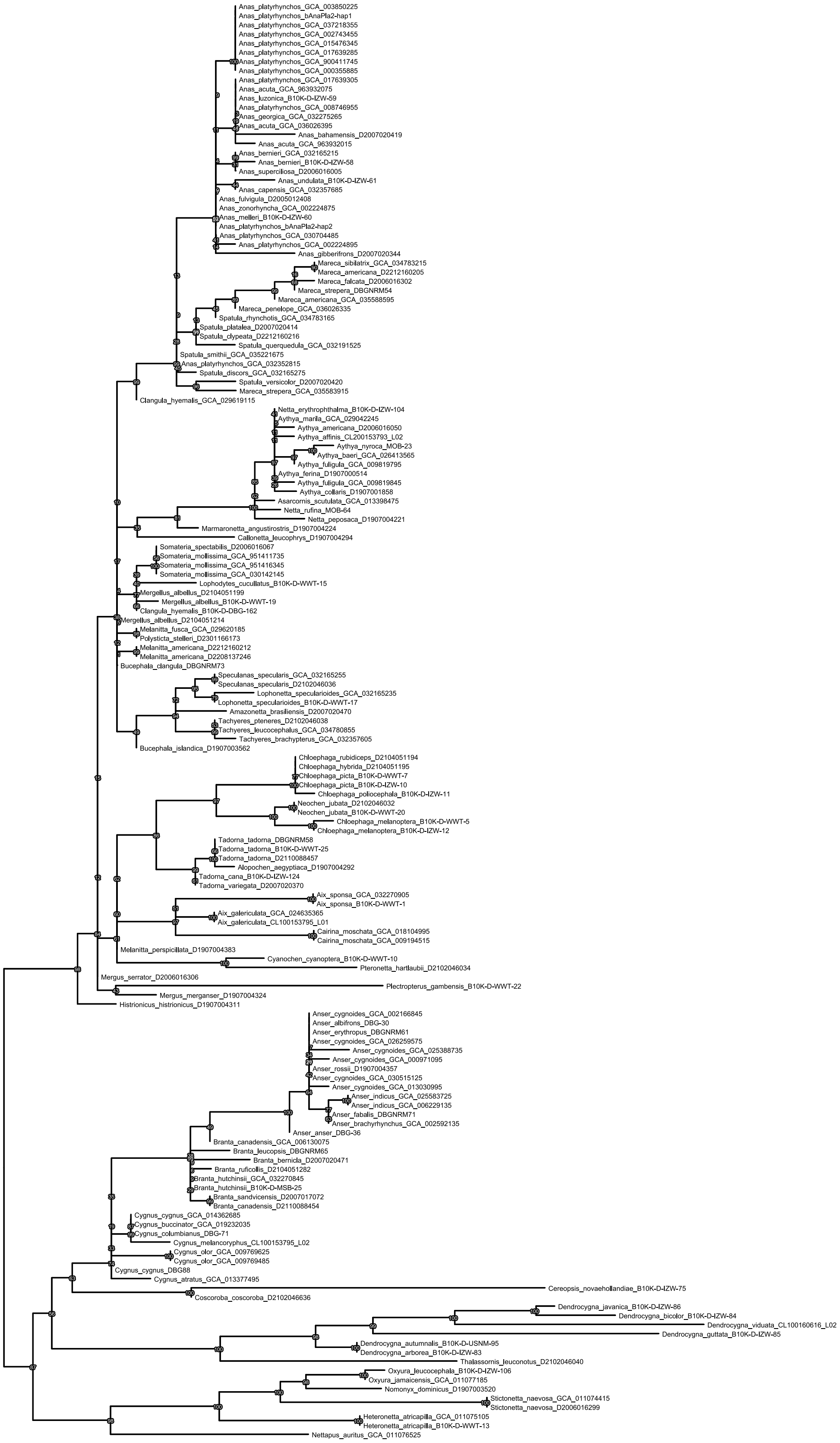

Supplementary Figure 4. Phylogenetic tree derived from 81 Clusterin sequences. The tree was rooted with the outgroup *Grus americana* (whooping crane), not shown in the plot. Bootstrap resampling support shown for all nodes. Source data: Zenodo[1].

Clusterin

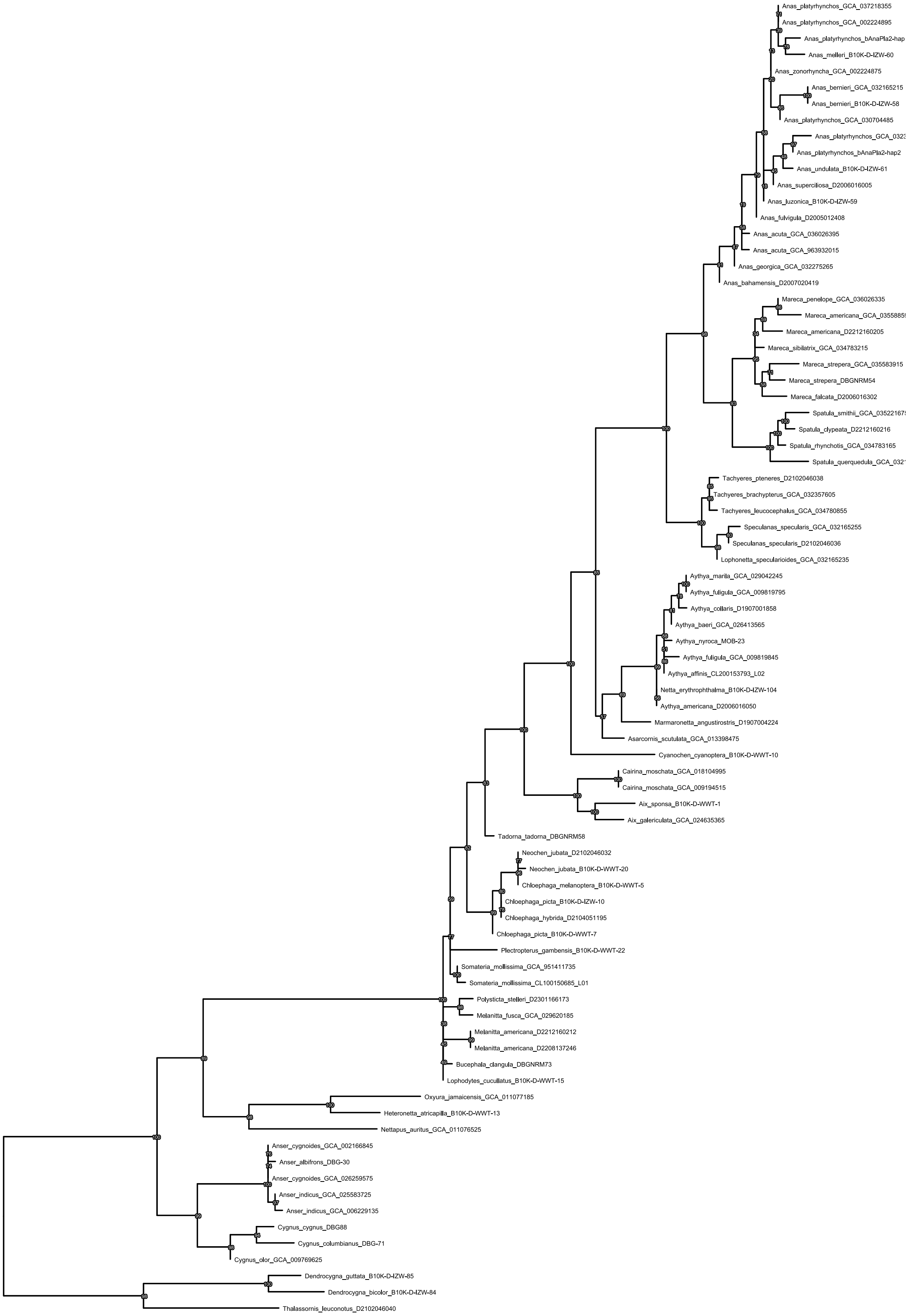

Supplementary Figure 5. Phylogenetic tree derived from 83 COL1a1 sequences. The tree was rooted with the outgroup *Grus americana* (whooping crane), not shown in the plot. Bootstrap resampling support shown for all nodes. Source data: Zenodo[1].

COL1a1

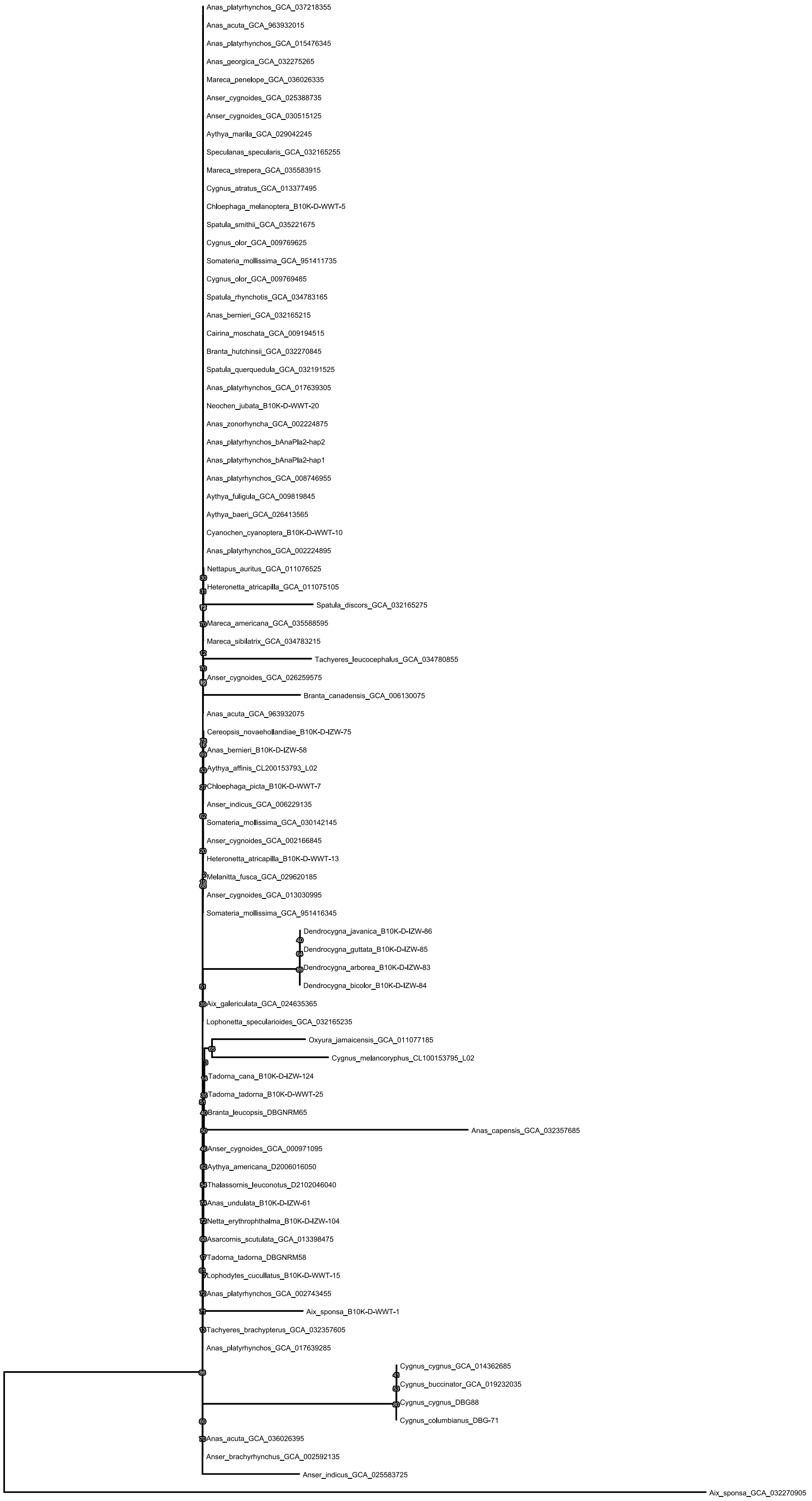

Supplementary Figure 6. Phylogenetic tree derived from 156 COL1a2 sequences. The tree was rooted with the outgroup *Grus americana* (whooping crane), not shown in the plot. Bootstrap resampling support shown for all nodes. Source data: Zenodo[1].

COL1a2

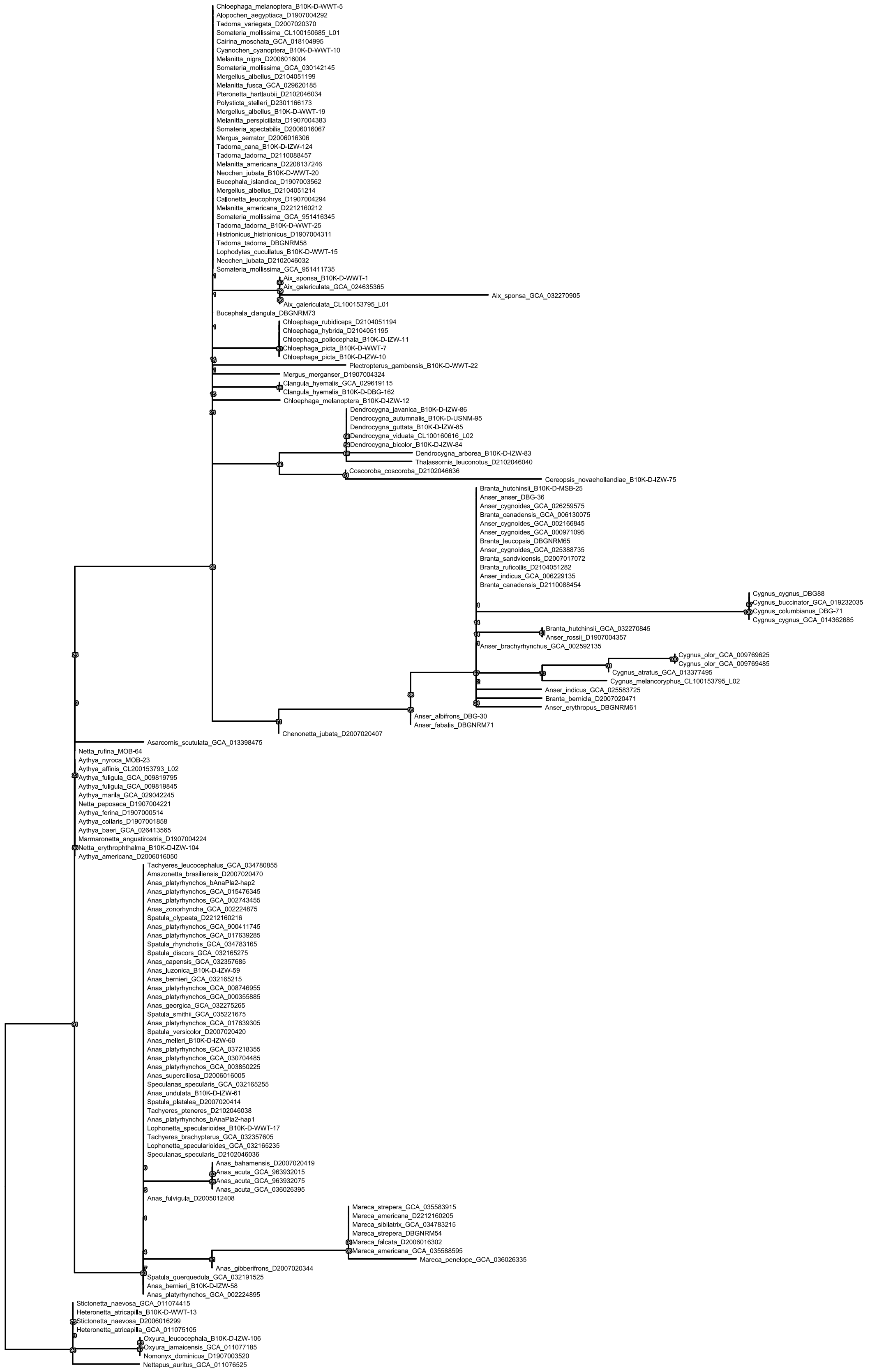

Supplementary Figure 7. Phylogenetic tree derived from 157 Lactadherin sequences. The tree was rooted with the outgroup *Grus americana* (whooping crane), not shown in the plot. Bootstrap resampling support shown for all nodes. Source data: Zenodo[1].

Lactadherin

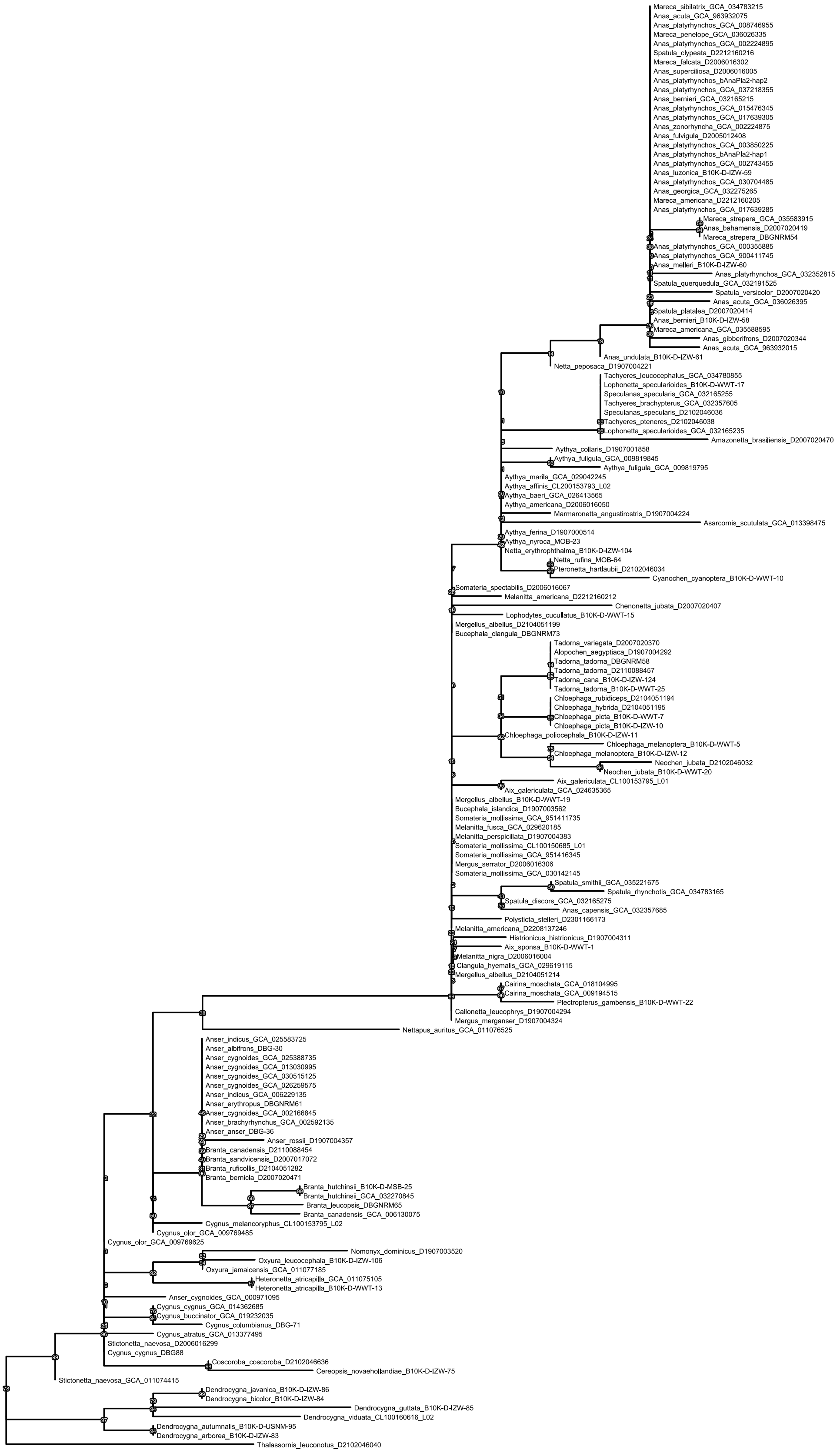

Supplementary Figure 8. Phylogenetic tree derived from 158 OC116 sequences. The tree was rooted with the outgroup *Grus americana* (whooping crane), not shown in the plot. Bootstrap resampling support shown for all nodes. Source data: Zenodo[1].

OC116

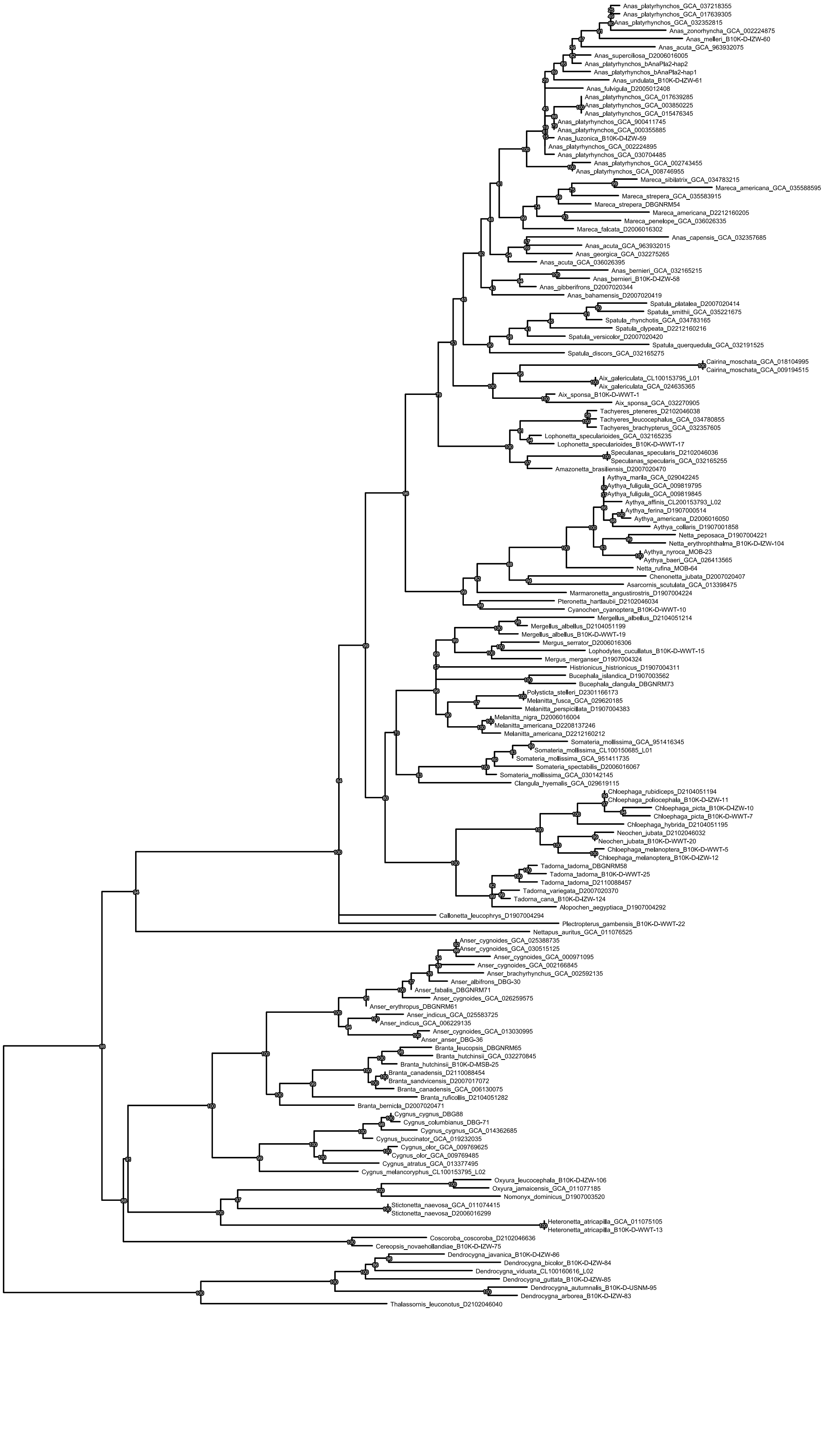

Supplementary Figure 9. Phylogenetic tree derived from 157 Ovalbumin sequences. The tree was rooted with the outgroup *Grus americana* (whooping crane), not shown in the plot. Bootstrap resampling support shown for all nodes. Source data: Zenodo[1].

Ovalbumin

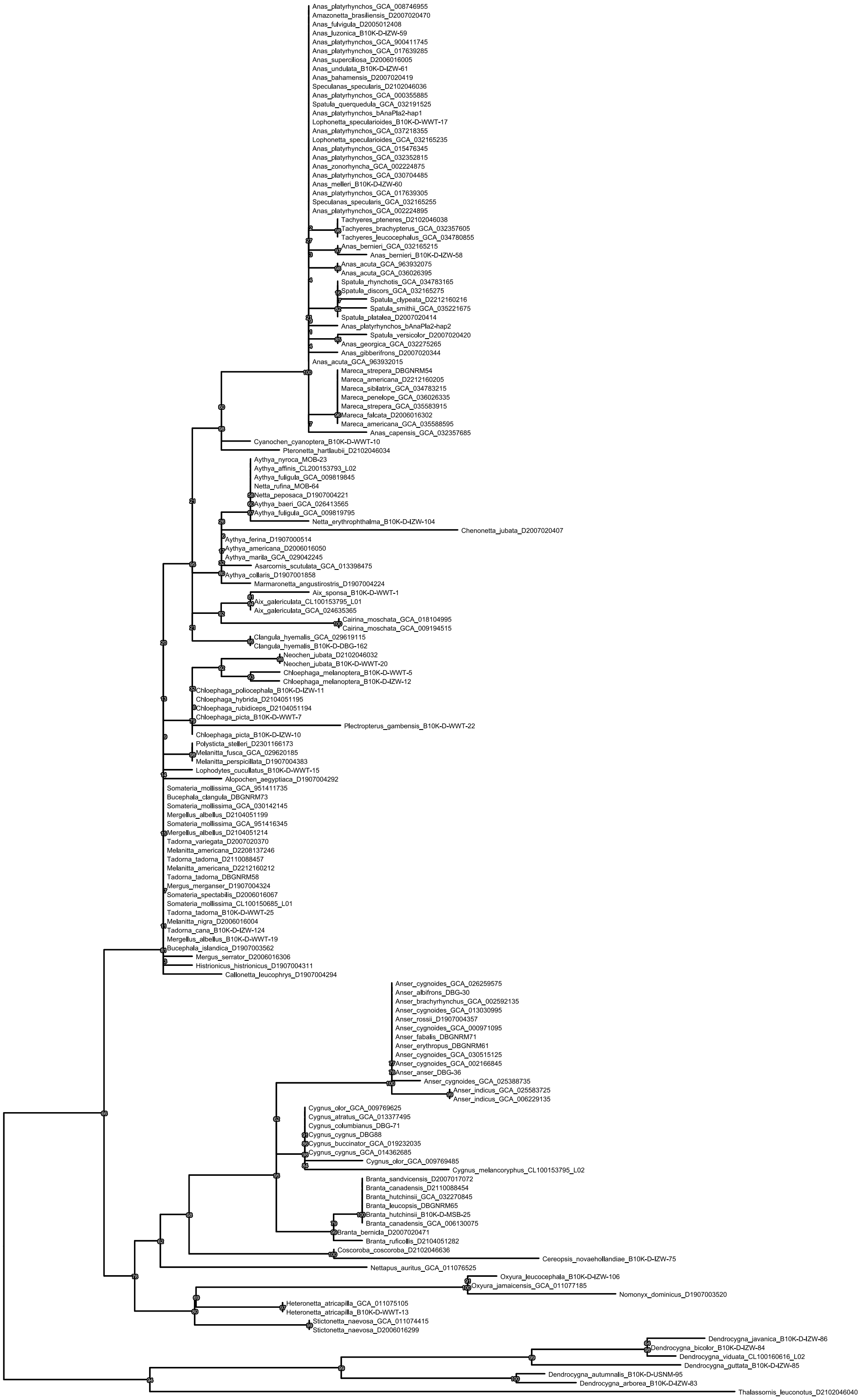

Supplementary Figure 10. Phylogenetic tree derived from 156 Ovocalyxin32 sequences. The tree was rooted with the outgroup *Grus americana* (whooping crane), not shown in the plot. Bootstrap resampling support shown for all nodes. Source data: Zenodo[1].

Ovocalyxin32

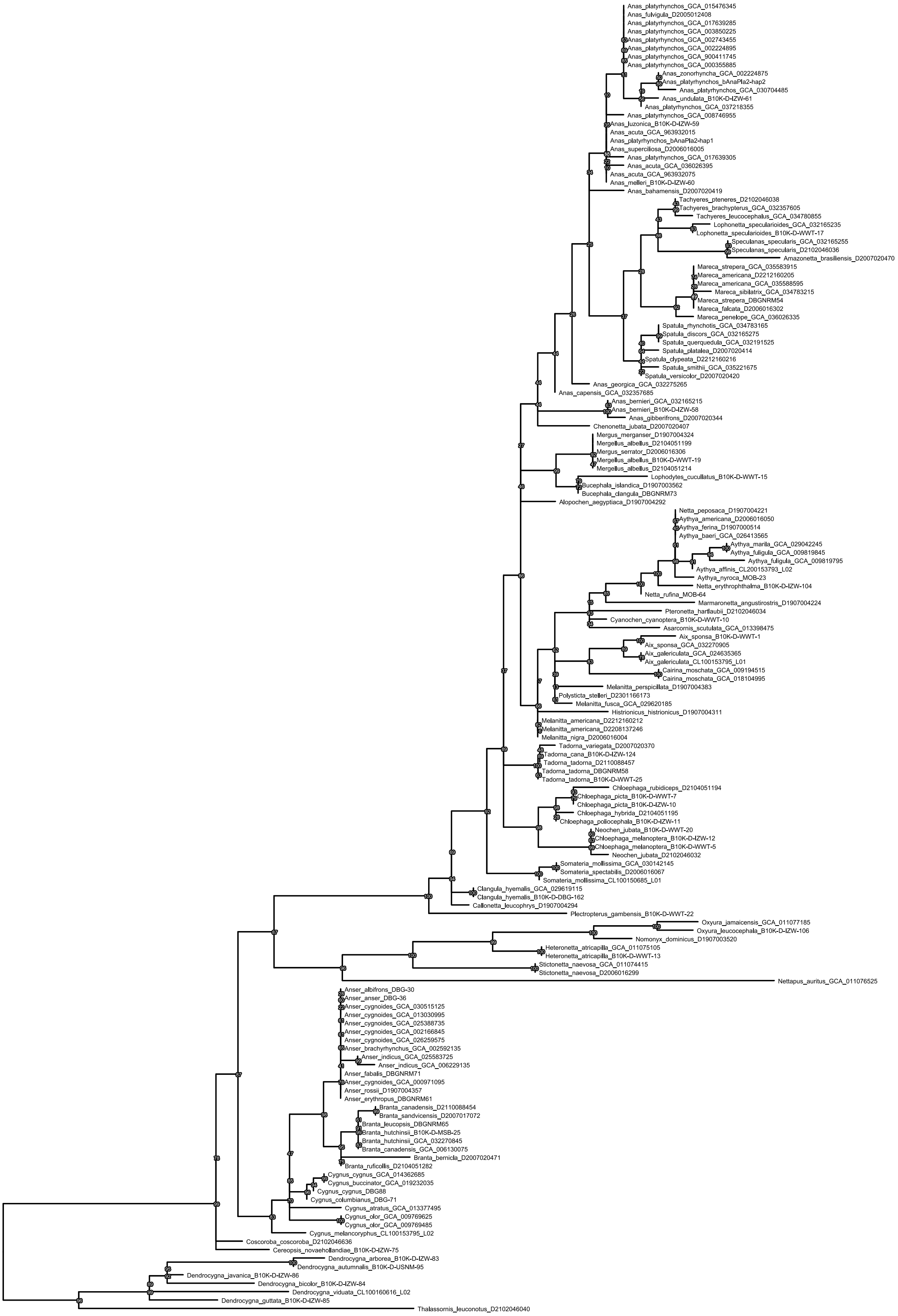

Supplementary Figure 11. Phylogenetic tree derived from 158 Ovomucoid sequences. The tree was rooted with the outgroup *Grus americana* (whooping crane), not shown in the plot. Bootstrap resampling support shown for all nodes. Source data: Zenodo[1].

Ovomucoid

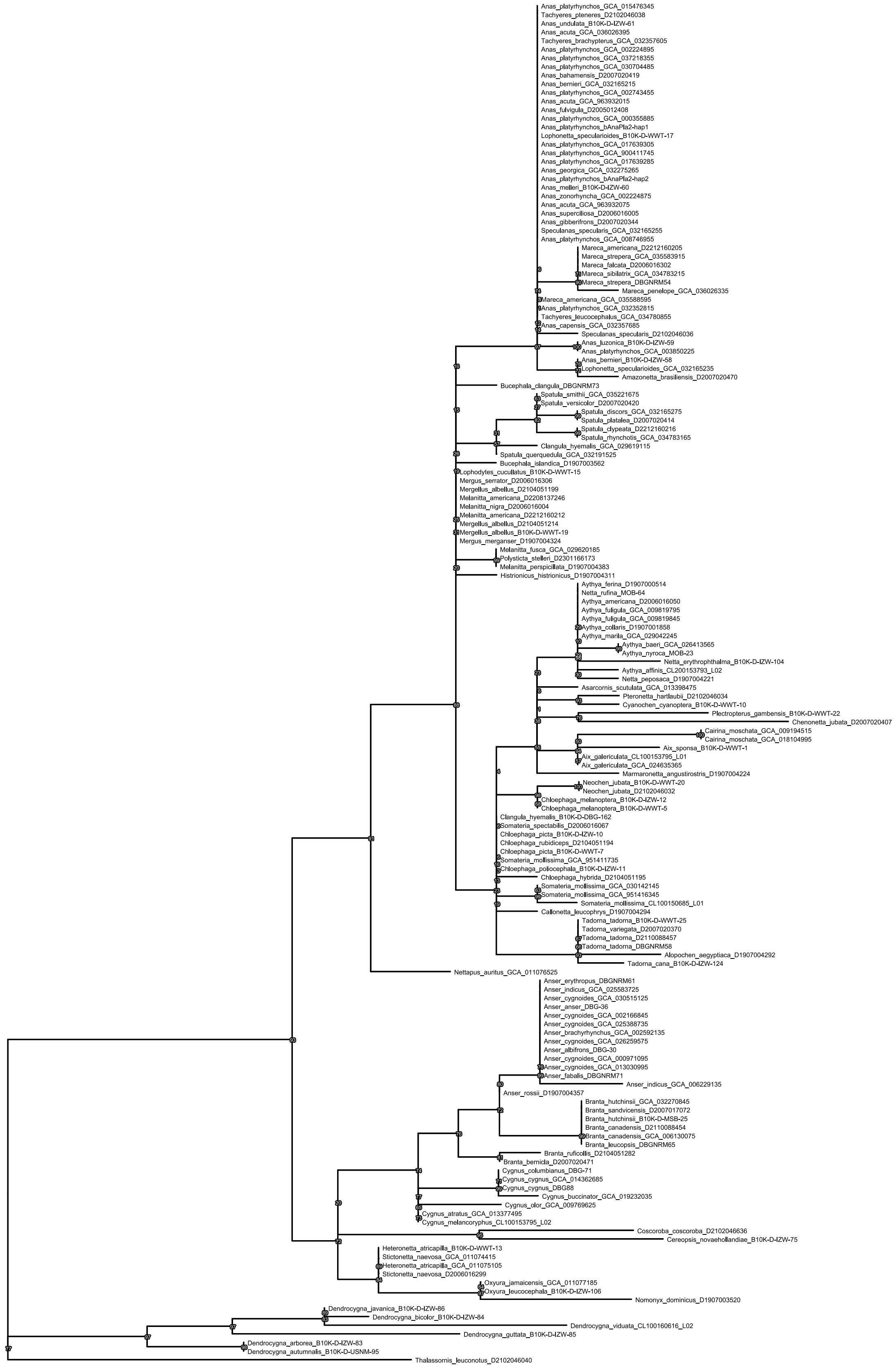

Supplementary Figure 12. Phylogenetic tree derived from 157 Ovotransferrin sequences. The tree was rooted with the outgroup *Grus americana* (whooping crane), not shown in the plot. Bootstrap resampling support shown for all nodes. Source data: Zenodo[1].

Ovotransferrin

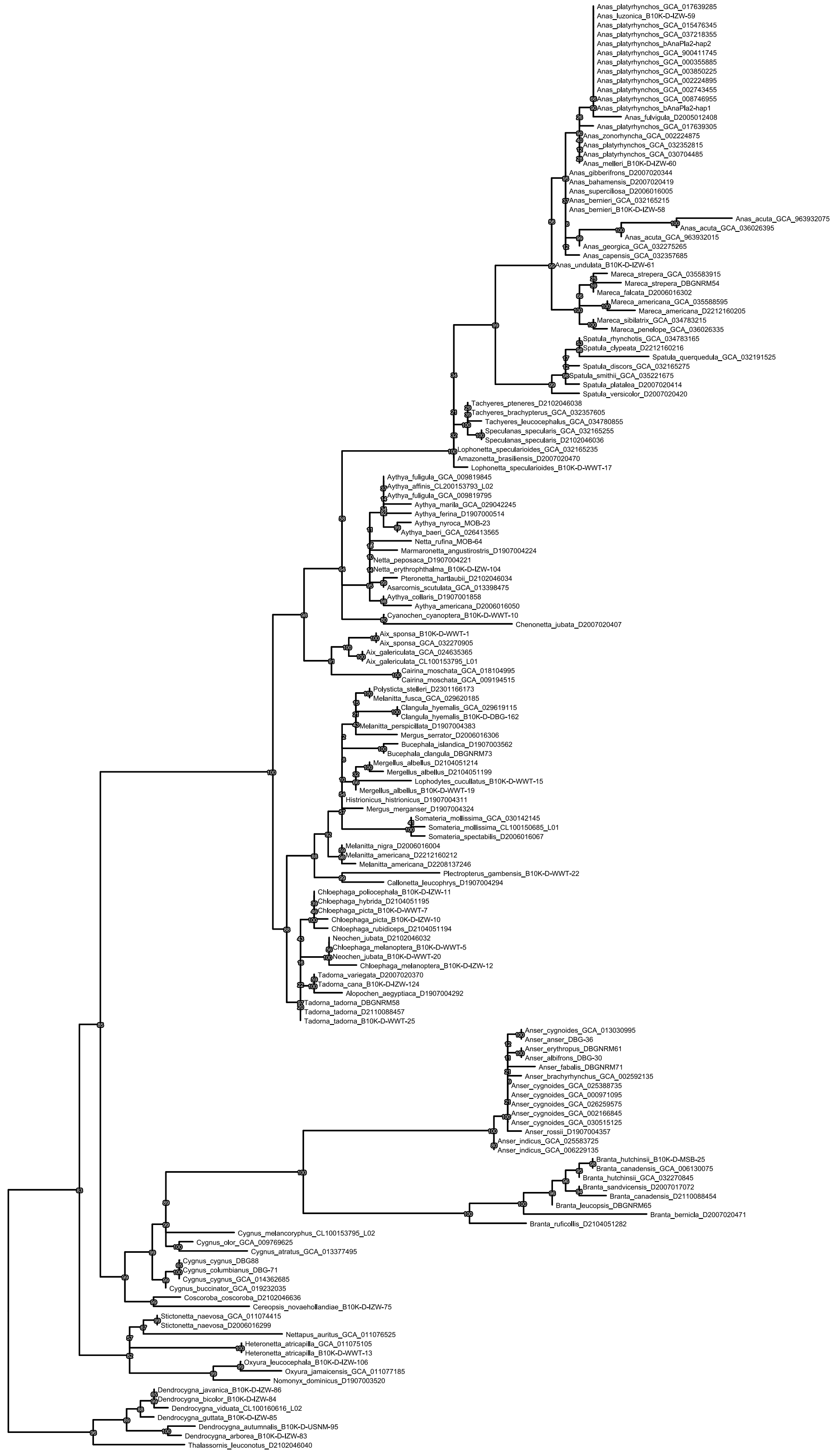

Supplementary Figure 13. Phylogenetic tree derived from 112 XCA1 sequences. The tree was rooted with the outgroup *Grus americana* (whooping crane), not shown in the plot. Bootstrap resampling support shown for all nodes. Source data: Zenodo[1].

XCA1

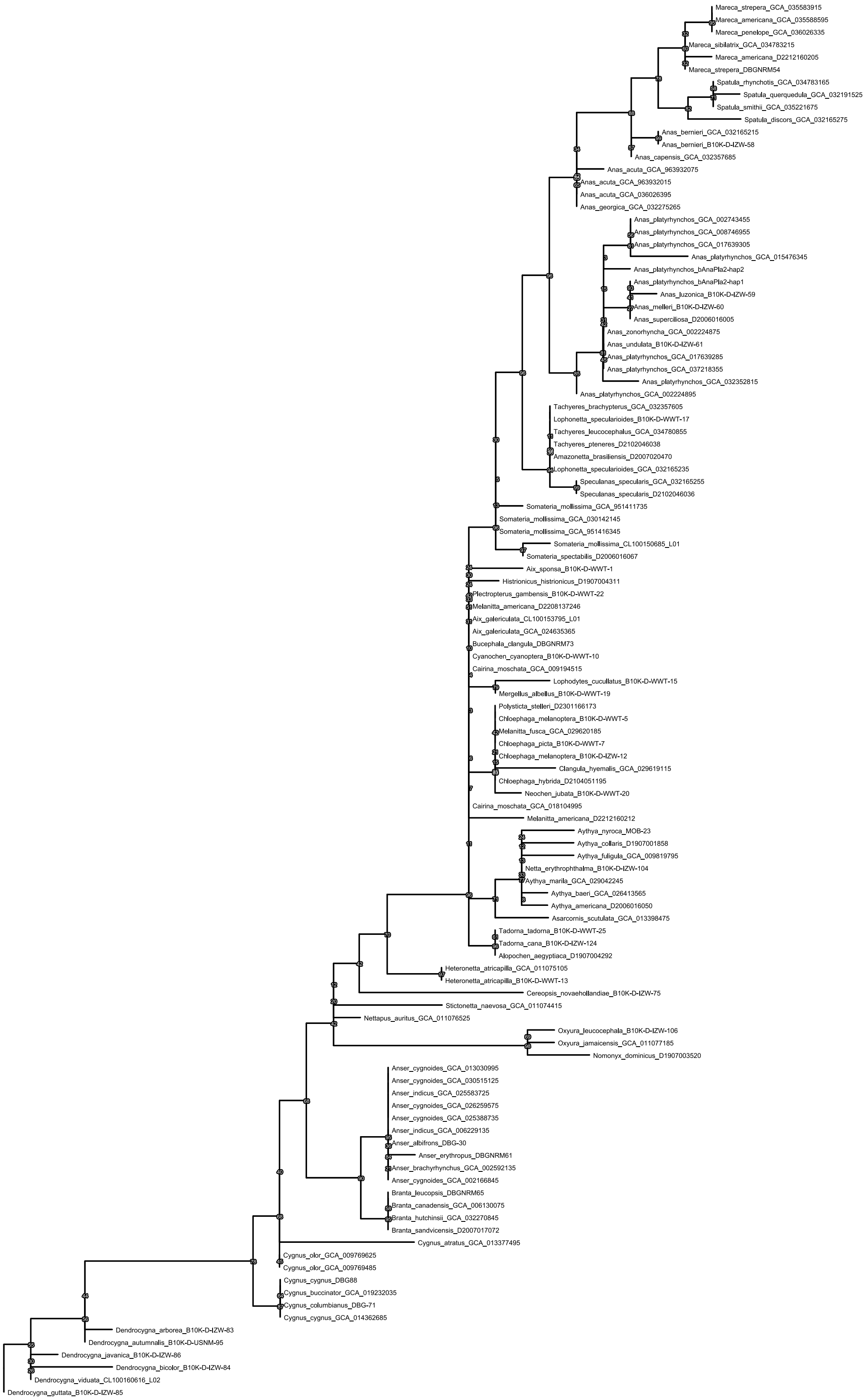

Supplementary Figure 14. Phylogenetic tree derived from 140 XCA2 sequences

XCA2

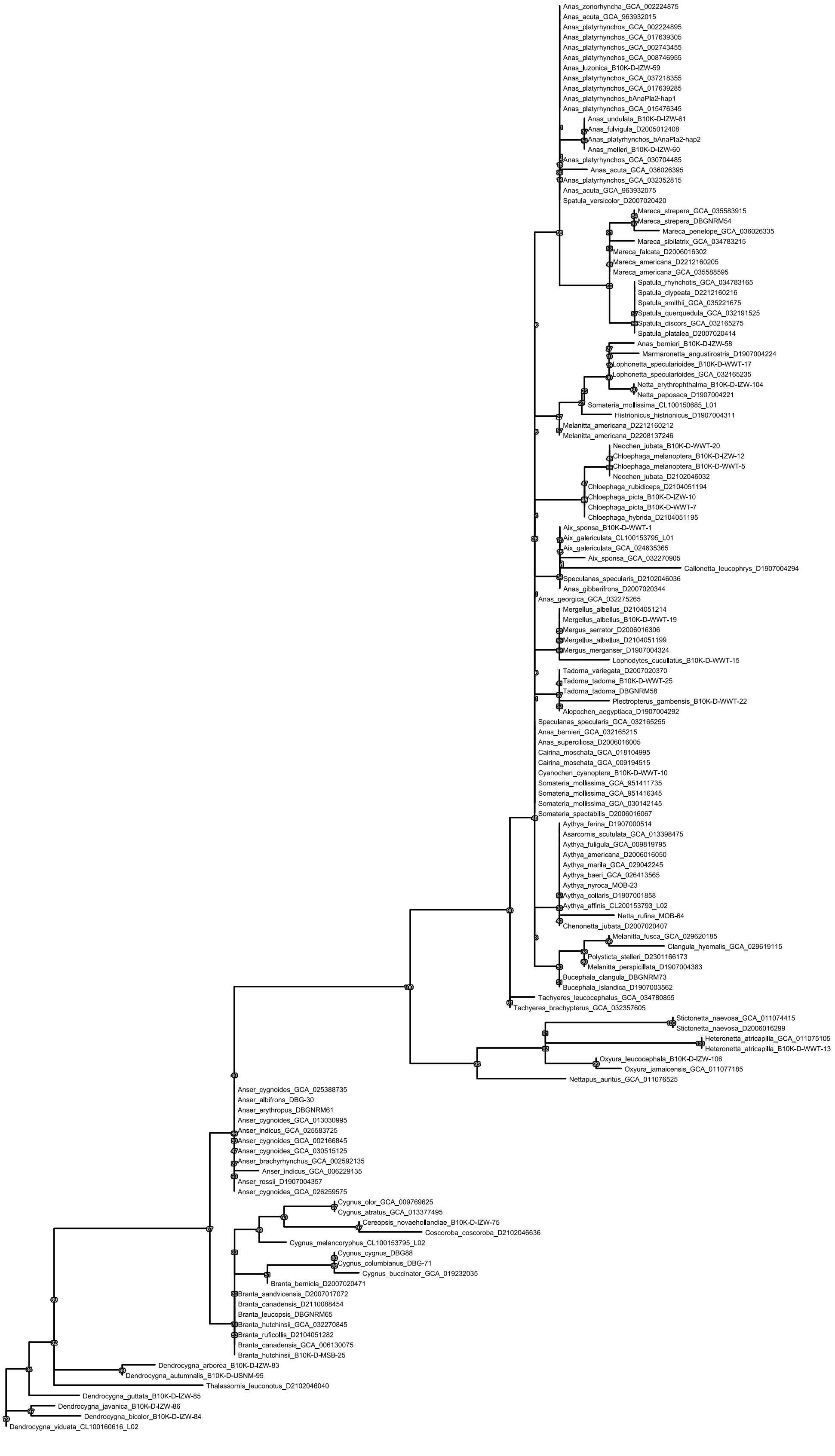

Supplementary Figure 15. Alignment of 14 OC116 sequences for *Anas platyrhynchos* demonstrating location of SAPs. SAPs with no confirmation through variant calling are highlighted in red. One SAP supported by variant calling is not observed in the alignment, but its position is indicated in green.

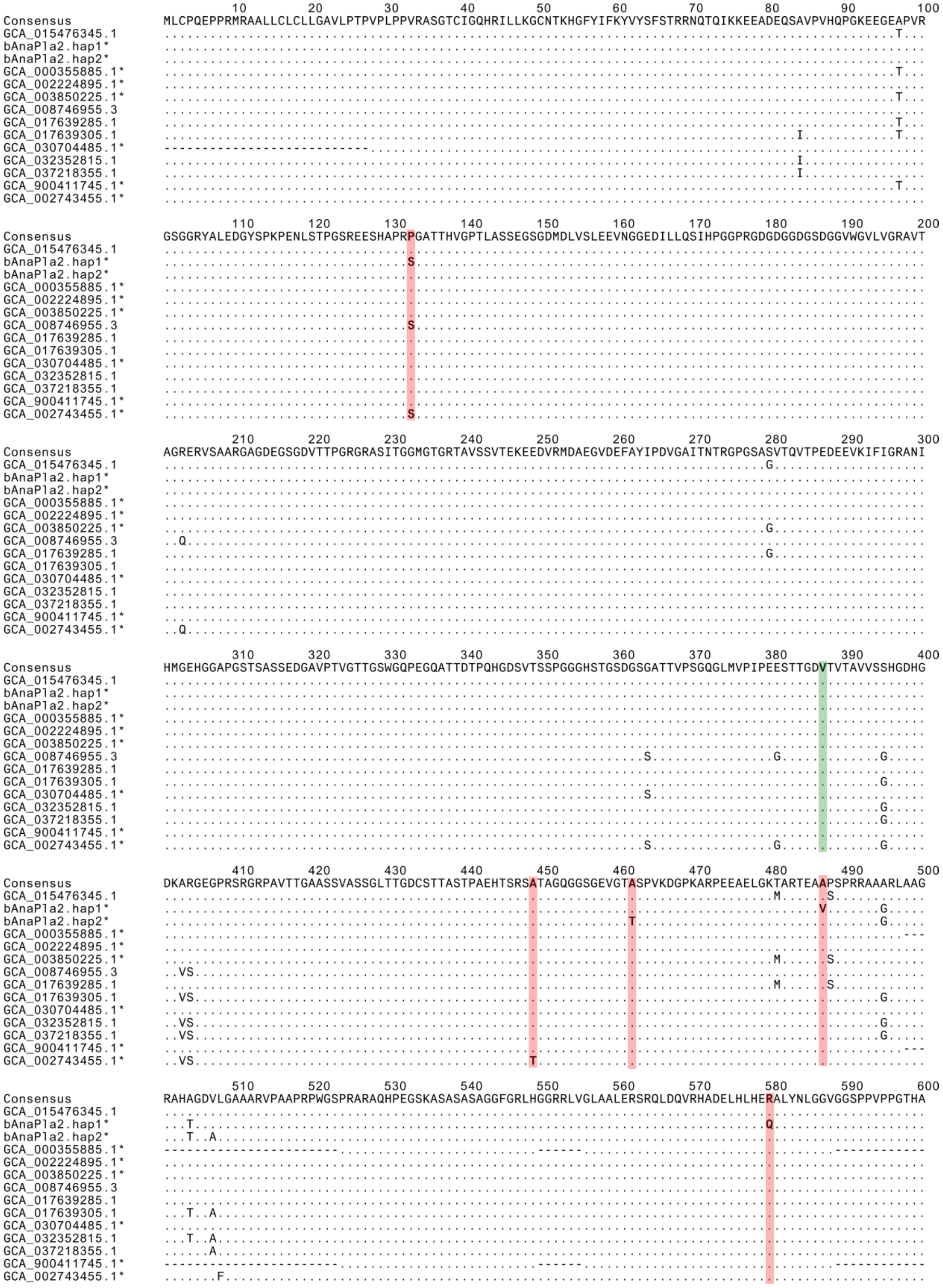

Supplementary Figure 16. Summary of protein alignments and selected spectra of peptide fragmentation (MS2s) that support identifications of *Cygnus olor* and *Anser* ssp.

```
                                    XCA2 consensus from pos.63-113                          OC116 consensus from pos. 180-199
                                    FGSGTHLASVHSEEELQAXVDLLFSSRSSDASEEELDEEVWIGLHRPLRSR     GDSDGGDGGDGGVWGVLVDR
Cygnus_atratus_GCA_013377495.2      ..................V...........................P....     ............A.......
Cygnus_buccinator_GCA_019232035.1   ..................M................................     ....................
Cygnus_columbianus_DBG-71           ..................M................................     ....................
Cygnus_cygnus_DBG88                 ..................M................................     ....................
Cygnus_melancoryphus_CL100153795_L02 ----------------..V................................     ....................
Cygnus_olor_GCA_009769625.2         ..................V...........................P....     ............G.......
```

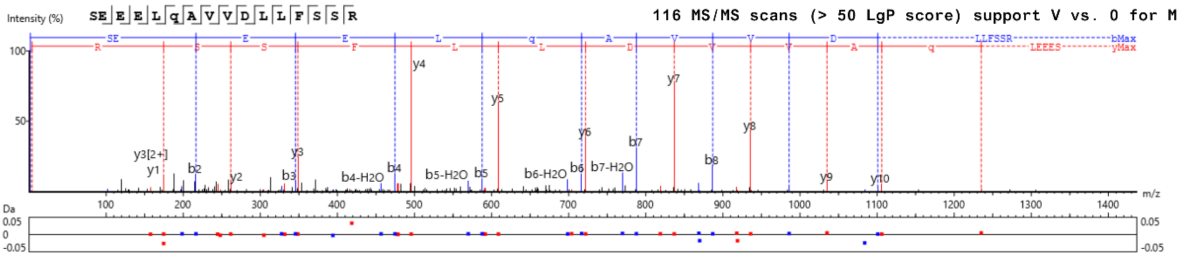

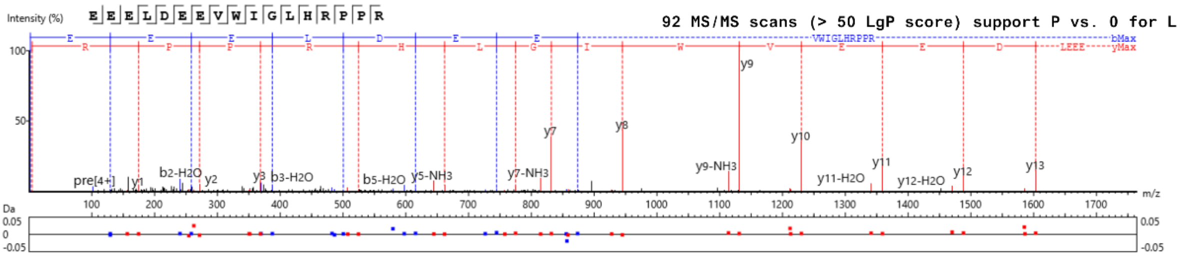

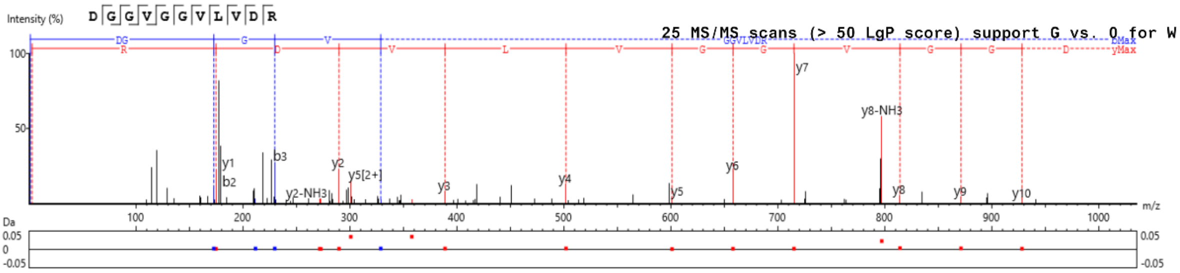


```
                                        XCA2 consensus from pos. 0-39              BPI-fold-B4 consensus from pos. 364-378
                                        MAPTWTPGLWLLGCLLLVPALWGECRRELSDCTPGWVPVSG  SVMIQVFVKRLDGSIINLLLLKADLSLNVHVSI:
Branta_bernicla_D2007020471             ........................---------------..  .........................I.......
Branta_canadensis_GCA_006130075.1       ..........................Q..............  .................................
Branta_hutchinsii_GCA_032270845.1       ..........................Q..............  .................................
Branta_leucopsis_DBGNRM65               ..........................Q..............  .................................
Branta_ruficollis_D2104051282           ..........................Q..............  .................................
Branta_sandvicensis_D2007017072         ..........................Q..............  .................................
Anser_albifrons_DBG-30                  .........................................  ...............L.V...............
Anser_cygnoides_GCA_002166845.1         .........................................  ...............L.V...............
Anser_erythropus_DBGNRM61               .........................................  ...............L.V...............
Anser_indicus_GCA_025583725.1           .........................................  ...............L.V...............
Anser_brachyrhynchus_GCA_002592135.1    .........................................  ...............L.V...............
```

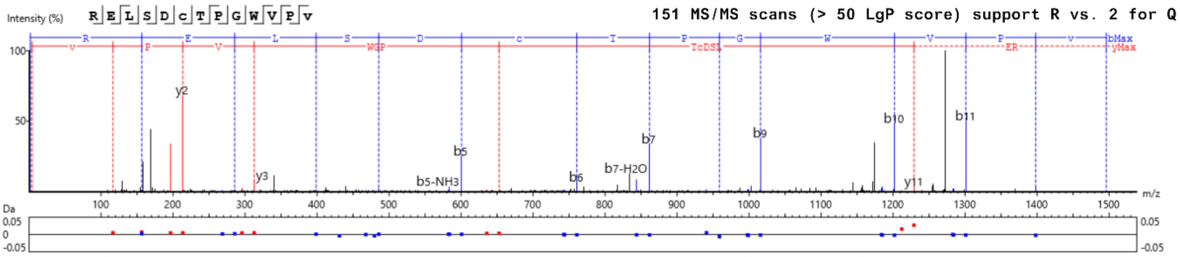

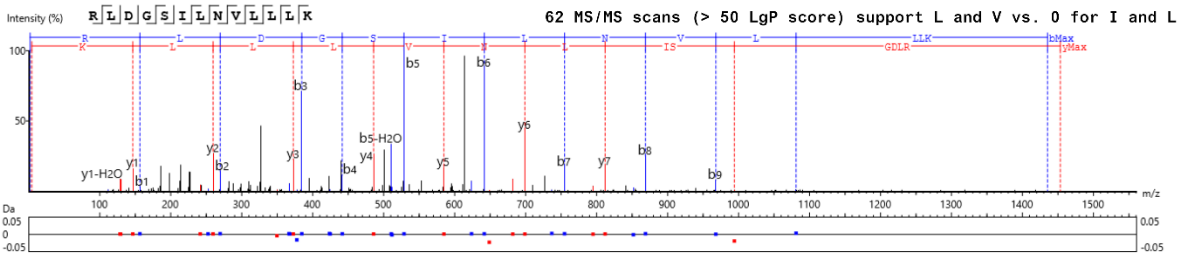

Supplementary Figure 17. Alignment of select ovotransferrin sequences annotated using the TRFE_ANAPL (*Anas platyrhynchos*) and XP_040423237.1 (*Cygnus olor*) references. Major deviations from the consensus are highlighted in yellow. See Supplementary Note 4

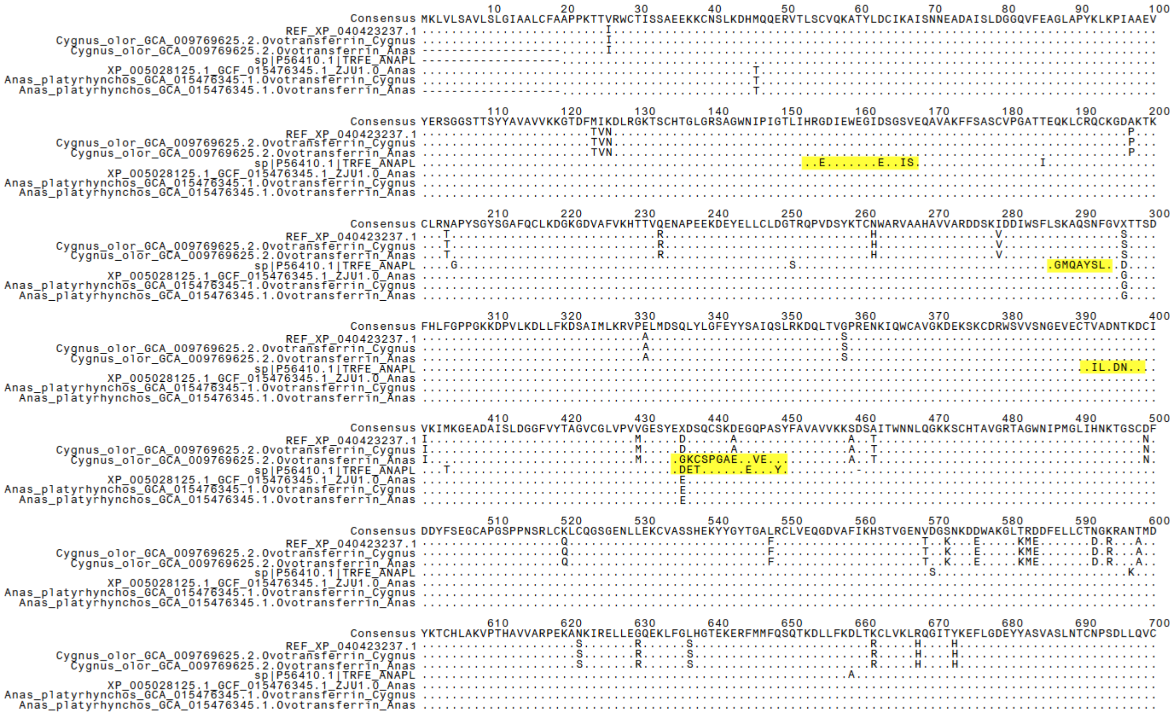

Supplementary Tables

Supplementary Table 1. Summary short-read samples available for genomes used in analysis and heterozygosity observed in missense SNPS. SRR11915221 and SRR11910010 represent separate individuals contributing to a pooled genome assembly, while SRR18178819 and SRR18186809 represent separate runs of a single sample.

SRR ID	Sample tissue	Biosample number	Instrument	Associated Genome	Associated genome notes	Het[1]
SRR25181664*	male muscle	SAMN36329575	Illumina HiSeq X	GCA_030704485.1	Monoisolate; male muscle	X*
SRR11915221	female liver	SAMN15090836	Illumina HiSeq X	GCA_015476345.1	Pooled individuals and tissues	N
SRR11910010	male muscle	SAMN15090885	umina HiSeq X			N
SRR12316517	female muscle	SAMN15638146	Illumina HiSeq 2000	GCA_017639285.1	Pooled females and tissues	N
SRR13051620	female muscle	SAMN15638092	Illumina HiSeq 2000	GCA_017639305.1	Pooled females and tissues	Y
SRR13076878	female muscle	SAMN15638089	Illumina HiSeq 2000	GCA_008746955.3	Pooled females and tissues	Y
SRR18178819	male blood	SAMN24405268	Illumina HiSeq 2500	GCA_037218355.1	Monoisolate; male blood and liver	Y
SRR18186809			Illumina HiSeq 2500			
SRR26345462	female blood	SAMN36468273	Illumina NextSeq 2000	GCA_032352815.1	Monoisolate; female blood	N

*sample SRR25181664 was removed from analysis following the unexplained presence of triploid allele variation in the *OC116* gene.

Het[1]: Heterozygosity observed

Supplementary Table 2. Summary of *OC116* reads mapped to the haploid resolved genome assemblies (bAnaPla2.hap1 and bAnaPla2.hap2). Ambiguous mapping is indicated by mapping quality scores (MQ) of 3 or lower and implies that a read maps to more than one location on the genome

	**Total *OC116* reads**		**# reads with ambiguous mappings**		**% ambiguous mappings**	
SRA ID	Hap 1	Hap 2	Hap 1	Hap 2	Hap 1	Hap 2
SRR11910010	3788	3788	120	137	3%	4%
SRR11915221	3068	3069	2	7	0%	0%
SRR12316517	916	911	23	25	3%	3%
SRR13051620	1892	1878	110	101	6%	5%
SRR13076878	2424	2419	69	61	3%	3%
SRR18178819	714	714	5	2	1%	0%
SRR18186809	703	703	0	0	0%	0%
SRR26345462	97	97	0	0	0%	0%

Supplementary Table 3. Biomarkers for four duck groups identified by Codlin et al[2] and discussed in Supplementary Note 3.

Duck group	ID	$[M+H]^+$	Peptide name	Peptide sequence
Anatidae 1	MC148	2777.3	COL1α2 454–483	GETGPAGPPGFQGLPGPSGPAGEAGKPGER
Anatidae 1	MC148	2985.5	COL1α2 757–789	GPSGESGAPGPPGTPGPQGILGAPGILGLPGSR
Anatidae 1	MC148	1632.8	COL1α2 889–906	GDPGPAGHVGPAGAFGPR
Anatidae 2	MC123	2804.3	COL1α2 454–483	GEQGPAGPPGFQGLPGPSGPAGEAGKPGER
Anatidae 2	MC123	2969.5	COL1α2 757–789	GPSGEAGAPGPPGTPGPQGILGAPGILGLPGSR
Anatidae 2	MC123	1632.8	COL1α2 889–906	GDPGPAGHVGPAGAFGPR
Anatidae 3	MC182	2804.3	COL1α2 454–483	GEQGPAGPPGFQGLPGPSGPAGEAGKPGER
Anatidae 3	MC182	2984.5	COL1α2 757–789	GPSGEAGAQGPPGTPGPQGILGAPGILGLPGSR
Anatidae 3	MC182	1632.8	COL1α2 889–906	GDPGPAGHVGPAGAFGPR
Oxyura (Anatidae 4)	MC171	2804.3	COL1α2 454–483	GEQGPAGPPGFQGLPGPSGPAGEAGKPGER
Oxyura (Anatidae 4)	MC171	2927.5	COL1α2 757–789	GPSGEAGAAGPPGTPGPQGILGAPGILGLPGSR
Oxyura (Anatidae 4)	MC171	1660.8	COL1α2 889–906	GDPGPVGHVGPAGAFGPR

Supplementary Table 4. Summary of new taxonomic identification of eggshell samples from Tlajinga, Teotihuacan and Shubayqa. Species in bold are considered the most likely.

Tlajinga, Teotihuacan, Mexico: COL1a2		
**Previous identification (representative specimen)**	**New taxonomic identification**	**Possible species present**
Anatidae Group 1 (MC148)	*Mareca*	*M. strepera, M. americana*
Anatidae Group 2 (MC123): Matched *Anas platyrhynchos* reference	*Anatini*<br>*Aythya*	*Anas diazi, A. platyrhynchos, A. fulvigula, A. crecca; Spatula discors, S. cyanoptera, S. clypeata; Aythya valisineri, Ay. americana, Ay. collaris, Ay. marila*
Anatidae Group 3 (MC182)	*Anas acuta*<br>*Anas bahamensis*<br>*Anas georgica*	***Anas acuta,*** *A. bahamensis*
Anatidae Group 4 (MC171): Matched *Oxyura jamaicensis* reference	*Oxyura*	***Oxyura jamaicensis***
**Shubayqa, Jordan: XCA1, XCA2, OC116 & BPI-fold-B4**		
**Previous identification (representative specimen)**	**New taxonomic identification**	**Possible species breeding**
Anatidae (PALTO 114D)	*Tadorna*<br>*Alopochen*	***Tadorna tadorna, T. ferruginea***<br>*Alopochen aegyptiaca*
Anatidae (PALTO 119D)	*Tadorna*<br>*Alopochen*	***Tadorna tadorna, T. ferruginea***<br>*Alopochen aegyptiaca*
Cygnus sp. (PALTO 689)	*Cygnus olor*	***Cygnus olor***
Anser/*Branta* (PALTO 693)	*Anser*	***Anser anser,*** *A. albifrons, A. erythropus*

Supplementary Table 5. List of proteins used as reference queries for annotation of genomes.

Accession ID	Protein	Description	Species
XP_035424404.1	COL1a1	collagen alpha-1(I) chain isoform X3	*Cygnus atratus*
NP_001383551.1	COL1a1	COL1A1	*Gallus gallus*
XP_038029841.1	COL1a2	collagen alpha-2(I) chain isoform X2	*Anas platyrhynchos*
XP_066846641.1	XCA1	rheacalcin-1-like	*Anser cygnoides*
XP_038024161.1	XCA2	struthiocalcin-2-like	*Anas platyrhynchos*
XP_038034818.1	OC116	ovocleidin-116 isoform X1	*Anas platyrhynchos*
NP_001297323.1	Albumin	serum albumin precursor	*Anas platyrhynchos*
XP_021122208.1	BPI-fold-B4	BPI fold-containing family B member 4	*Anas platyrhynchos*
XP_032041356.1	Clusterin	clusterin	*Aythya fuligula*
AAD17257.1	Clusterin	clusterin	*Gallus gallus*
XP_027321929.1	Lactadherin	lactadherin isoform X1	*Anas platyrhynchos*
NP_001298098.1	Ovalbumin	ovalbumin	*Anas platyrhynchos*
XP_035422339.1	Ovocalyxin 32	retinoic acid receptor responder protein 1	*Cygnus atratus*
XP_035415626.1	Ovomucoid	ovomucoid	*Cygnus atratus*
XP_040423237.1	Ovotransferrin	ovotransferrin	*Cygnus olor*

Supplementary Note 1: Assessment of annotation quality

We compared protein sequences from our study to proteins annotated by NCBI's RefSeq annotation pipeline: GCF_015476345.1 (*Anas platyrhynchos*), GCF_009819795.1 (*Aythya fuligula*), GCF_009769625.2 (*Cygnus olor*), GCF_013377495.2 (*Cygnus atratus*), and GCF_011077185.1 (*Oxyura jamaicensis*). For most proteins, no, or minor differences were observed (Supplementary Data 6). Minor differences include partial annotations or inclusion of segments in the RefSeq sequences that appear erroneous, often at the N or C termini. In many cases, annotation was likely similar across both pipelines, but erroneous segments were removed during our manual data curation.

Potentially meaningful differences were identified in three proteins: XCA1, XCA2 and COL1a2. For XCA1, *A. platyrhynchos* annotations varied at amino acids 120-122 (ungapped), where the three-letter sequence was "LPX" in the RefSeq sequence and "-PQ" in the sequence annotated by our pipeline. In contrast, the protein sequence was annotated as "LPR" in seven of eight other *A. platyrhynchos* specimens. This implies that this difference is likely an isolated incident, such as one caused by sequencing or assembly error or that annotations were performed on different versions of the assembled genome.

For COL1a2, amino acids at positions 25-28 varied from the RefSeq annotations for *A. platyrhynchos* and *A. fuligula* ("VSE" to "LFQ"), and at 24-34 for *C. atratus* and *C. olor* ("HVSEAPAGRR" to "RKLLSACSWP") and *O. jamaicensis* ("HVSEAPAGRR" to "RKLLSSVVVQ"). These variants are internally consistent within the rest of the COL1a2 dataset, with most ducks presenting the LFQ variant, and geese, swans and stiff-tailed ducks presenting the longer variant.

For XCA2, a three amino acid insertion ("ECR") at position 24 in *C. atratus* deviates from the ReqSeq sequence. In the XCA2 dataset, this insertion is found consistently in geese, swans and whistling ducks. Moreover, the insertion means that this section of the protein aligns more closely with other Anatidae species, as it would otherwise be a gap in the sequence.

Given the consistency in COL1a2 and XCA2 deviations from the RefSeq sequences, there is insufficient evidence to indicate they are erroneous annotations. Moreover, as they are located near the N-terminus, these parts of the sequences are unlikely to be incorporated into the final protein product. Based on this, we decided to not to trim or modify these portions of the sequences.

Supplementary Note 2: Comparison of annotated *Anas platyrhynchos* sequences and SRA support for OC116 SAPs

A total of 19 SAPs were identified across 14 *Anas platyrhynchos* sequences for OC116 (Supplementary Figure 15). Nine Sequence Read Archives (SRA) were available for seven of the genomes to evaluate the support for these SAPs (Supplementary Table 1). Of the 19 SAPs, 14 were supported by variant calling analysis and 4 SAPs (at 449, 462, 487, and 580) were not evaluated because they derived from genomes not included in the SRA data for variant calling. One SAP at position 133 was not well supported by variant calling, as it had low read coverage (n=4) in the sample with this variant. However, it is likely that this variant does exist in the *Anas platyrhynchos* population given that it was also observed in two other sequences where SRAs were not available for variant calling (Supplementary Figure 15). One mutation supported by variant calling (at 387) was observed only in the SRA data as the annotated genome from this sequence was assembled from multiple individuals.

Supplementary Note 3: Taxonomic identification of specimens at Tlajinga and Shubayqa

The annotated protein sequences comprising the reference dataset (dataset 2) were applied to previously analysed bone and eggshell samples to assess the improvement in taxonomic resolution with the new dataset. We use n^g, n^{sp}, and n^i to refer to the number of distinct genera, species and individual sequences respectively. Where the number of individual sequences and the number of individual samples differ, as in the case of phased genomic sequences, this is specifically clarified in text. Protein-based identification techniques usually involve one or two types of mass spectrometry. Matrix-assisted laser desorption/ionisation time-of-flight (MALDI-TOF) mass spectrometry is a cheap yet relatively low resolution technique that provides, peptide mass-fingerprint, a spectrum of peaks characteristic of the mass of peptides within a sample. In contrast, tandem mass spectrometry techniques (MS/MS) provide higher resolution as they not only measure the mass of a peptide, but fragment it further to reconstruct the sequence of amino acids in the peptide.

Anatidae at Tlajinga, Teotihuacan

Our estimates of what might have been present are conservative and account for the fact that our current information about species distribution in Mexico mostly derives from studies that occurred after major infilling of the large and diverse lake system that before European contact was a major wintering ground for North American waterfowl[3]. Therefore, we consider it possible that any species with a current distribution in or near Mexico are potential candidates, but provide an estimate for the most likely species based on commonly occurring species in the region today. Of 173 species in Anatidae (as listed in the HBW and BirdLife International 2024 checklist[4] we have the COL1a2 sequence for 111 species providing excellent coverage of species found in this region.

Codlin et al.[2] presented LC-MS/MS data to support multiple MALDI-TOF biomarkers (i.e. peptide sequences and mass-to-charge ratios) which were observed to distinguish four groups of specimens identified to Anatidae (Supplementary Table 3). In their study, group 2 matched peptide sequences found in *Anas platyrhynchos* and group 4 matched peptide sequences found in *Oxyura jamaicensis*. The filtered protein and peptides from our PEAKS 11 analysis of the four specimens from these groups supported the peptide sequences and masses identified by Codlin et al.[2].

Anatidae group 1 made up 11 % of the Anatidae identified at Tlajinga, Teotihuacan. The combination of peptides that peak at *m/z* 2777.3 and 2985.5 were found only in species from the *Mareca* genus (n^{sp}=5), and were observed in all individuals (n^i=7). Considering the modern distributions of *Mareca*, only *M. strepera* and *M. americana* are likely to have been present in Central Mexico in the past[5] (Supplementary Table 4). While there is a theoretical peptide found in many COL1a2 sequences with a predicted *m/z* = 2777.4, this peptide requires a missed trypsin cleavage and the mass is not observed in the spectra of other species that share this peptide sequence, including all Anatidae discussed here. The LC-MS/MS data for sample MC148 and non-Anatidae taxa from Codlin et al. (2022) with spectra presenting a peak at *m/z* 2777.3 confirms that the mass derives from the peptide sequence predicted by the COL1a2 sequences for *Mareca*.

Anatidae group 2 was the largest group in the Anatidae assemblage (56%) and the combination of biomarkers representative of this group (*m/z* 1632.8, 2804.3 and 2969.5), are found in multiple genera (n^{g}=28). The filtered results of the PEAKS analysis of the LC-MS/MS data identified close matches in COL1a2 to species in two groups visible in the COL1a2 tree (Supplementary Figure 6), one containing *Amazonetta* (n^{sp}=1), *Anas* (n^{sp}=10), *Lophonetta* (n^{sp}=1), *Spatula* (n^{sp}=7), *Speculanas* (n^{sp}=1) and *Tachyeres* (n^{sp}=3), and the second containing *Aythya* (n^{sp}=8), *Marmonetta* (n^{sp}=1), and *Netta* (n^{sp}=3). These two groups can be distinguished by a T to A SAP at position 576, but this portion of the COL1a2 sequence was not recovered by the LC-MS/MS analysis. Of these species, those that could have lived or over-wintered in Central Mexico[5] include *Anas diazi, A. platyrhynchos, A. fulvigula, A. crecca* (COL1a2 sequence unknown), *Spatula discors, S. cyanoptera, S. clypeata, Aythya valisineri, Ay. americana, Ay. collaris*, *Ay. marila* and *Netta erythrophthalma*. Given the broad range of species in this group, the size and shape of bones of these specimens could be employed to further narrow down this identification.

In group 3 (18%), a combination of markers was observed at *m/z* 2804.3, 2984.5 and 1632.8. The *m/z* 2984.5 peak is unique within dataset 2 (i.e. no other tryptic COL1a1 or COL1a2 peptide has this mass) and this peptide is only found in pintails: *Anas acuta* (three sequences from two individual specimens) and *A. bahamensis* (n^{i}=1). One SAP separates these species from other *Anas* ssp., but given that it is present in four pintail genomes, and no other species, we consider the SAP as a marker for pintail taxa. A third pintail species, *A. georgica*, does not present this SAP, but given that only one individual was sequenced, we cannot rule out that this SAP may be present in the *A. georgica* population. However, only *Anas acuta* visits this region today[5] and is therefore the best candidate for taxonomic identification.

Anatidae 4 was tentatively identified as *Oxyura jamaicensis* by Codlin et al.[2], as the markers matched the theoretical peptides from an annotated *Oxyura jamaicensis* genome and proteins from two reference specimens available for this species. *Oxyura* (n^{sp}=2, n^{i}=2) is the only genus with the peptides or masses at *m/z* 1660.8, 2927.5, and 2804.3, and although *Nomonyx dominicus* (n^{i}=1) has an identical pairwise identity, it is missing coverage of the sequence across two of these peptides. Of the two *Oxyura*, only *O. jamaicensis* would have been present in Mexico in the past and is common today, while *N. dominicus* could potentially have been present in the past.

In three out of four of these duck groups, taxonomic identification can be made based on MALDI-TOF MS data alone, while group 2 requires multiple other biomarkers best observed with LC-MS/MS data.

Anatidae at Shubayqa, Jordan

We also applied our new dataset to improve taxonomic resolution of eggshells recovered from Shubayqa, a Late Pleistocene to Early Holocene transition site in Eastern Jordan. Yeomans et al.[6] suggested that most eggshell samples belong to a species of duck for which protein sequences were not available. They also identified evidence of goose (*Anser/Branta*), and one swan (*Cygnus*).

With the larger dataset, we can confirm that the unidentified duck with a distinctive marker at *m/z* 2461.2 belonged to a species not present in the original database. *Tadorna* (n^{i}=4, n^{sp}=2), *Alopochen* (n^{i}=1) and *Plectropterus* (n^{i}=1) all have the SAP in the XCA2 sequence which results in the marker at *m/z* 2461.2. Therefore they cannot be distinguished by MALDI-MS. However, there are 36 SAPs in OC116 and 18 SAPs in BPIfoldB4 that separate the single *Plectropterus* genome from the consensus sequence of *Tadorna* (n^{i}=5, n^{sp}=3). For each of these two proteins, we found that 7-50% more peptides matched to *Tadorna* than to *Plectropterus* (Supplementary Tables 26 and 29), hence we are confident in ruling out *Plectropterus*. Only one *Alpochen* genome was available, and the lower quality of this genome means that fewer proteins were successfully annotated for this species, making it difficult to

rule out based on protein sequence information. Today, *Alopochen* is considered an introduced species in Jordan, while two species of *Tadorna* are naturally occuring[7,8]. We cannot rule out a change in the breeding and distribution ranges of *Alopochen* and *Tadorna ferruginea* from the Late Pleistocene to today, however, given that *Tadorna tadorna* occasionally breeds in the region[9,10] and specimens from this genus were identified in the osteological assemblage from Shubayqa[6], *Tadorna* sp. is the best candidate for the late Pleistocene breeding population.

The LC-MS/MS data for the sample previously identified at *Cygnus* sp. matched most closely to protein sequences found in *Cygnus olor* (Supplementary Tables 3-4) . *Cygnus olor* diverged from other swans found in the northern hemisphere, *C. cygnus*, *C. columbianus* and *C. buccinator* around 7.5 mya (according to Sun et al.[11]), which is reflected as SAP differences between these species in every protein in our study with the exception of Ovalbumin (see Supplementary Figures 2-14). While only one *C. olor* individual (two sequences) is present in our database, there are seven individuals in total for *the Cygnus* genus (n^{sp}=6). The PEAKS data for the archaeological sample identified 72 peptide matches to *C. olor,* compared to 56 to *C. cygnus* for OC116, and 58 peptide matches to *C. olor* compared to 28 to *C. buccinator, C. cygnus* and *C. columbianus*. Moreover, while all three *C. olor* SAPs identified in the archaeological XCA2 sequence are also found in *C. atratus* (n^{i}=1), this species is native to Australia. Overall, this suggests that many of the SAPs identified in the archaeological sample are not the result of intraspecies variation, supporting the identification as *C. olor* (Supplementary Figure 16). Today, all *Cygnus* species, including *C. olor*, are considered rare or accidental visitors to Jordan. According to Hansson et al.[12], the first published and confirmed sighting of *Cygnus olor* in Jordan was in January ’98, four swans in the Azraq wetland. The archaeological eggshell fragments present clear evidence that mute swans breed in Jordan at the end of the Pleistocene and start of the Holocene.

Multiple consistent SAPs in both XCA1 and XCA2 proteins support the separation of *Anser* and *Branta* geese (Supplementary Figure 16). The goose specimen from Shubayqa displays amino acid sequences found in the *Anser* genus for these proteins, including 35 peptides mapping only to *Anser* XCA1 peptides and 23 mapping only to *Anser* XCA2 according to the PEAKS algorithm (Supplementary Table 35). . While the archaeological sample has 146 peptide matches to the OC116 sequence from *A. cygnoides* (GCA_013030995.1) compared to the next highest hit (136) to *A. indicus* (GCA_006229135.1), the intraspecies variation observed in OC116 for both species prevents us from more precisely identifying this specimen until interspecies variability identified in OC116 can be distinguished from intraspecies. *Anser anser* is a winter visitor to Jordan today, while *A. albifrons* and *A. erythropus* are rare or accidental visitors[13]. Based on current phylogeography, it is therefore more likely that *Anser anser* was breeding in Jordan during the late Pleistocene.

Supplementary Note 4: Selection of reference proteins (queries)

For more variable proteins, using reference proteins from closely related species, in this case, from the same family, usually provided better coverage and accuracy of retrieved sequences than sequences from less related taxa. In other cases, we found that using two reference sequences, one closely related and one less closely related, such as *Gallus gallus*, and then merging the resulting annotations prior to the creation of the consensus sequence improved annotated sequences over using a single reference. Where possible, we used reviewed SWISS-PROT proteins[14] or RefSeq annotated proteins[15] for *Anas platyrhynchos* genome (GCF_015476345.1). Often, however these annotations were missing or inconsistent with other annotations from closely related species, and so sequences matching a wider consensus were chosen.

Reference sequences for collagen type 1 (COL1a1 and COL1a2), c-type lectins (XCA1 and XCA2), Ovocleidin 116 (OC116), albumin, BPI fold containing family B member 4 (BPI-fold-B4, BPIFB4), clusterin, lactadherin, ovalbumin, ovocalyxin32, ovomucoid, and ovotransferrin are listed in Supplementary Table 5.

COL1a1 was originally annotated using XP_035424404.1 (*Cygnus atratus*) as the reference sequence. It was noted, however, that average coverage of the resulting proteins was poorer than during preliminary testing using NP_001383551.1 (*Gallus gallus*). The cause of this is unclear, given the highly conserved nature of COL1a1. We additionally ran the annotation pipeline using the *Gallus* reference for any genome where the initial consensus sequence was less than about 70% coverage, and the results of both annotation runs were aligned together to create the consensus sequence. The resulting merged dataset had higher coverage of the protein overall than either dataset individually. For the same reason, the clusterin protein was also annotated using two reference proteins AAD17257.1 (*Gallus gallus*) and XP_032041356.1 (*Aythya fuligula*) and the results merged as above.

During the first round of annotations, the reviewed SWISS-PROT protein P56410.1 TRFE_ANAPL for *Anas platyrhynchos* was chosen as the target reference for ovotransferrin. However, when comparing annotations based on this protein against the RefSeq annotations, we observed that proteins for *A. platyrhynchos* and many other species diverged significantly from the RefSeq proteins (Supplementary Figure 17). Annotation of ovotransferrin was instead run using *Cygnus olor* XP_040423237.1, which while longer than the accepted protein length[16,17], included the complete ovotransferrin protein.

Supplementary Note 5: Description of additional data available on Zenodo

Additional files are uploaded to the following public repository:

Codlin, M. C., & Stiller, J. (2025). Genome annotation pipeline and library of sequences for 13 bone and eggshell proteins for Anatidae (ducks, geese and swans) [Data set]. Zenodo. https://doi.org/10.5281/zenodo.16932720

Annotation pipeline:

Readme_Annotation.md
- Provides notes for setup to run the annotation pipeline

Demo.zip
- This contains a copy of the .readme file and annotation pipeline scripts, one genome (GCA_015476345.1 downloaded from NCBI 21/05/2025) and 2 queries to demo the tool. Also includes an example output for comparison.

queryseg.sh
- Bash script which creates segments of queries prior to running Annotation pipeline

Codlin_et_al_Annotation_pipeline.sh
- Bash script for the annotation pipeline to extract protein sequences from genomes

Queries.zip
- This contains 15 .fa files, each containing a single protein sequence used as a reference query for annotating Anatidae proteins. Files have been named with protein_Genus.fa for use in the annotation pipeline indicating the genus related to the reference protein.

Complete dataset.zip
- *Curated sequences*
    - 13 .fasta file alignments for each protein, containing complete and partial curated sequences, all replicates.
    - Each sequence in this file is named with a code designating the first three letters of the Order and Family of the species (OrdFam code), followed by the species name and genome accession ID, and finally the name of the protein used as the query and a "consensus" if the sequence was derived from alignment of multiple fragments. i.e. AnsAna_Anas_acuta_GCA_963932075.Albumin_Anas.consensus
- *Geneious format annotation files*
    - 13 .geneious file alignments for each protein, containing complete and partial curated sequences, all replicates. All modifications made during curation are recorded using Geneious Prime 2024.0 as annotations. These files can be viewed using the free version of Geneious available at (https://www.geneious.com)
- *Unmodified alignments of sequence fragments*
    - 13 .fasta file alignments for each protein containing bulk alignment of all annotated proteins and protein segments created by annotation pipeline. No modifications were made to these sequences except alignment. This file was used to evaluate sequences during curation and identify erroneous SAPs. Sequences are named with the OrdFam code, the species name, genome accession ID, the query protein, and the annotation information indicating scaffolds and locations from the genome where the protein sequences were extracted from.

Dataset 1.zip
- 13 .fasta file alignments for each protein, containing all curated sequences which were at least 70% complete

- Sequence names have been modified slightly compared to the complete curated sequences alignment. Each sequence is named with the OrdFam code, the species name, genome accession ID, the protein and "curated_70"

Dataset 2.zip

- 13 .fasta file alignments for each protein, containing both partial and complete curated sequences with redundancy removed at the species level.

Assessment of annotation quality

Annotation_RefSeq_alignments.zip

- Contains 13 aligned fasta files comparing proteins annotated by NCBI's pipeline for five Anatidae RefSeq genomes, and the sequences produced in this study for the same genomes.

SRA analysis and variant calling

Aplat_SRA_mapped_variant_calling_OC116.zip

- *SRA_and_variant_calling_commands.md*
    - Contains chunks of command line code used to create files in this .zip folder, including mapping Anas platyrhynchos SRAs to the OC116 gene, examining mapping quality as a marker of gene duplication and performing variant calling.
- 8 .bam files (and .bai index files) of *Anas platyrhynchos* SRAs mapped to the *Anas platyrhynchos* gene for OC116. These are labelled with the SRA ID and the filename of the gene used for alignment i.e. SRRXXXXX.NC_051775.1_OC116_gene.bam (and .bai).
- NC_051775.1_OC116_gene.fasta is the OC116 gene used for alignment of reads.
- Aplat_db.filtered.maf0.05.DP10.recode.vcf is the final combined variant calling file which incorporates the results from all 8 SRA files.

Aind_SRA_mapped_COL1a2.zip

- Contains one .bam file (and .bai index file) for the *Anser indicus* SRA mapped to the COL1a2 gene for *Anser cygnoides*. This is labelled with the SRA ID and the filename of the gene used for alignment i.e. SRR19551126.Anser_cygnoides_NC_088874.1_COL1a2.bam
- Anser_cygnoides_NC_088874.1_COL1a2.fasta is the COL1a2 gene used for alignment of reads.

Annotation of non-Anatidae:

queries_non_Anatidae.zip

- This contains 5 .fa files, each containing a single protein sequence used as a reference query for annotating non-Anatidae proteins. Files have been named with protein_Genus.fa for use in the annotation pipeline indicating the genus related to the reference protein.

Non_anatidae_OC116.fasta

- Alignment of OC116 protein sequences annotated from non Anatidae taxa

Non_anatidae_Ovocalyxin32.fasta

- Alignment of Ovocalyxin32 protein sequences annotated from non Anatidae taxa

Creation of phylogenetic trees

Trees.zip

- This file contains file IQTREE outputs from constructing phylogenetic trees from individual proteins and the concatenated proteins.

R code for producing figures and tables

Trees_fig1_fig2.Rmd

- R markdown notebook containing code used for production of base files for concatenated tree (Figure 1), individual trees showing only *Anas*, *Mareca* and *Spatula* used to make Figure 2, the full trees for each protein (Supplementary Figures 2-14) and the concatenated tree with full labels (Supplementary Figure 1).

Annotation summary.Rmd

- R markdown notebook file containing code used to create a summary of annotations in Dataset 1, Dataset 2 and the complete dataset by species (Supplementary Data 2).

DistancesVariation_fig3_fig4.Rmd

- R markdown notebook file containing code for pairwise distance analysis of protein sequences from Dataset 1, and for creating base files for Figures 3-4.

Reanalysis of LC-MS/MS data using PEAKS

Anatidae_DS2_SwissProtbird_validated.fasta

- Fasta file used as PEAK11 search database, include sequences from Dataset 2 alongside all bird proteins downloaded from SwissProt on 01/08/24

References

1. Codlin, M. C. & Stiller, J. Genome annotation pipeline and library of sequences for 13 bone and eggshell proteins for Anatidae (ducks, geese and swans). Zenodo https://doi.org/10.5281/zenodo.16932720 (2025).
2. Codlin, M. C., Douka, K. & Richter, K. K. An application of zooms to identify archaeological avian fauna from Teotihuacan, Mexico. *Journal of Archaeological Science* **148**, 105692 (2022).
3. Alcocer, J. & Williams, W. D. Historical and recent changes in Lake Texcoco, a saline lake in Mexico. *Int. J. Salt Lake Res.* **5**, 45–61 (1996).
4. HBW and BirdLife International. *Handbook of the Birds of the World and BirdLife International Digital Checklist of the Birds of the World. Version 8.1*. (2024).
5. *Birds of the World*. (Cornell Laboratory of Ornithology, Ithaca, NY, USA, 2022).
6. Yeomans, L., Codlin, M. C., Mazzucato, C., Dal Bello, F. & Demarchi, B. Waterfowl eggshell refines palaeoenvironmental reconstruction and supports multi-species niche construction at the Pleistocene-Holocene transition in the Levant. *J. Archaeol. Method Theory* **31**, 1383–1429 (2024).
7. Andrews, I. J. *The Birds of the Hashemite Kingdom of Jordan*. (1995).
8. Meinertzhagen, R. Ornithological results of a trip to Syria and adjacent countries 1933. *Ibis (Lond. 1859)* **13**, 110–151 (1935).
9. Wallace, D. I. M. The breeding birds of the Azraq Oasis and its desert surround, Jordan, in the mid-1960's. *Sandgrouse* **5**, 1–18 (1983).
10. Andrews, I. J. *The Birds of the Hashemite Kingdom of Jordan*. (1995).
11. Sun, Z. *et al.* Rapid and recent diversification patterns in Anseriformes birds: Inferred from molecular phylogeny and diversification analyses. *PLoS One* **12**, e0184529 (2017).
12. Hansson, E., Magnusson, A. & Eriksson, P. The first Mute Swan Cygnus olor and

Radde's Accentor Prunella ocularis in. *Sandgrouse* **20**, 46–47 (1998).

13. Andrews, I. J. *The Birds of the Hashemite Kingdom of Jordan*. (1995).
14. UniProt Consortium. UniProt: the Universal Protein Knowledgebase in 2025. *Nucleic Acids Res* **53**, D609–D617 (2025).
15. O'Leary, N. A. *et al.* Reference sequence (RefSeq) database at NCBI: current status, taxonomic expansion, and functional annotation. *Nucleic Acids Res* **44**, D733-45 (2016).
16. Wu, J. & Acero-Lopez, A. Ovotransferrin: Structure, bioactivities, and preparation. *Food Research International* **46**, 480–487 (2012).
17. Williams, J., Elleman, T. C., Kingston, I. B., Wilkins, A. G. & Kuhn, K. A. The Primary Structure of Hen Ovotransferrin. *European Journal of Biochemistry* **122**, 297–303 (1982).
